# Evaluation of high-throughput sequencing for replacing the conventional adventitious virus detection assays used for biologics

**DOI:** 10.1038/s41541-025-01351-2

**Published:** 2025-12-23

**Authors:** Pei-Ju Chin, Jen-Hui Tsou, Alison Armstrong, Noémie Deneyer, Valeria Zanda, Sophie Ayama-Canden, Anne-Sophie Colinet, Sandra M. Fuentes, Nikolay Korokhov, Christophe Lambert, Alfonso Lavorgna, Manuel Noll, Simone Olgiati, Michel Protz, Shahjahan Shaid, Afshin Sohrabi, Mary Whiteman, Arifa S. Khan

**Affiliations:** 1 Division of Viral Products, Office of Vaccines Research and Review, Center for Biologics Evaluation and Research, U.S. Food and Drug Administration, Silver Spring, MD USA; 2 Millipore Sigma, Rockville, MD USA; 3 GSK, Wavre, Wavre, Belgium; 4 EMD Serono RBM S.p.A. Istituto di Ricerche Biomediche A. Marxer, Via Ribes 1 10010 Colleretto Giacosa, Italy, an affiliate of Merck KGaA, Darmstadt, Germany; 5 Akkodis Belgium c/o GSK, Evere, 1140 Belgium; 6 GSK, Rixensart, Rixensart, Belgium; 7 GSK Marburg, Marburg, Germany; 8 Present Address: Office of the Director, Facility of Biotechnology Resources, CBER USA FDA, Silver Spring, MD USA; 9 Present Address: Integrative Biological Sciences (InBioS) Bât. B22 Integrative Biological Sciences, Liège 1, Belgium

**Keywords:** Biotechnology, Vaccines, Virology

## Abstract

High-throughput sequencing (HTS) can detect known and novel adventitious viruses in biological materials that might be missed by the conventional in vitro and in vivo assays. We compared HTS with the conventional assays using the same sample preparation of CHO cell harvest spiked with human respiratory syncytial virus (RSV) and mammalian orthoreovirus (Reo1). The viruses were selected with the expectation that one would produce a positive result in each test. The study results indicated both RSV and Reo1 were detected by HTS and in vitro assays, albeit with inter-assay and intra-lab differences in sensitivity of virus detection, and only Reo1 was detected by the in vivo assays. The results support using HTS to replace the in vivo assays. Additionally, based on capabilities of HTS for non-targeted virus detection, it may supplement or replace the in vitro assays. The study further highlighted that the analytical sensitivity of HTS can be improved.

## Introduction

Testing for viral adventitious agents is required for the safety of human and veterinary products. This involves using general and specific virus detection assays for testing samples involved in the manufacturing process, for example, the starting materials such as the cell bank, and viral vector preparation or vaccine virus seed, and the unprocessed drug substance (may also be referred to as the unprocessed harvest)^1–4^. The latter material is an intermediate to the final product and is a complex mixture that may contain varying amounts of host cell-derived components (such as whole or disrupted cells, nucleic acids, and proteins), in addition to the intended high-titer vaccine or vector virus, or high concentration of a recombinant protein product, which may negatively impact the assays for adventitious virus detection.

High-throughput or next-generation sequencing (HTS/NGS) has emerged as an advanced technology for broad virus detection. Unlike other advanced nucleic acid-based methods^5^, HTS can detect known and novel viruses without prior viral sequence information. However, the HTS workflow contains multiple steps, each of which can influence the breadth and sensitivity of virus detection^6,7^. This is particularly important for adventitious virus testing of different types of biological materials, particularly those intermediate to the final product. Furthermore, the success of broad virus detection by HTS relies on using a comprehensive, well-annotated reference virus database for interrogating the HTS data. With the recent publication of the guideline entitled “Q5A(R2) Viral Safety Evaluation of Biotechnology Products Derived from Cell Lines of Human or Animal Origin” prepared by the International Council for Harmonisation of Technical Requirements for Pharmaceuticals for Human Use (ICH)^8^, HTS is recognized as a potential broad virus detection technology for evaluating viral safety of biologics. The capabilities of HTS for broad adventitious virus detection were initially highlighted by the unexpected finding of porcine circovirus type 1 (PCV1) in a licensed rotavirus vaccine^9^ and by the discovery of a novel rhabdovirus in the insect *Spodoptera frugiperda* Sf9 cell line that is used for baculovirus-expressed products^10^. In both cases, extensive testing using the recommended in vitro and in vivo assays had demonstrated the absence of adventitious viruses^11,12^.

In this study, we have performed a direct comparison of HTS, in vitro cell culture assays, and in vivo animal assays for virus detection by spiking selected viruses in a complex matrix consisting of an unprocessed harvest material prepared from the Chinese hamster ovary (CHO)-K1 cell line, which is a common cell substrate for manufacturing of recombinant protein products^13^ and some vaccines^14^. RNA viruses were included to challenge the HTS technology and human respiratory syncytial virus (RSV) and mammalian orthoreovirus (Reo1) were specifically selected for the spiking study based on their expected detection of at least one virus in all the test methods, and particularly for sensitive detection in the in vivo and in vitro assays. Other viruses such as PCV1, Epstein-Barr virus (EBV) and feline leukemia virus (FeLV) known to be detected by HTS^15^ were not included since they would not be detected in the conventional assays. The results of our study support the recommendations in the ICH Q5A(R2) for considering HTS for replacing the in vivo assays for adventitious virus detection, and aligns with the global 3R’s initiative for replacing and reducing animals for testing^16,17^. Furthermore, based upon the demonstrated sensitivity for virus detection, HTS may be considered for supplementing or potentially replacing the in vitro assays. These results can facilitate decision-making about the applications of HTS for testing of biologics.

## Results

A collaborative spiking study was undertaken to compare the sensitivity of virus detection by HTS, in vitro cell culture assays, and in vivo animal assays. Three laboratories (designated as Lab 1, 2 and 3) performed HTS, Lab 2 and 3 also conducted the in vitro assays, and Lab 3 further conducted the in vivo assays. Two viruses, RSV and Reo1, were spiked into CHO-K1 cell-based unprocessed harvest material, which represented a complex matrix to mimic virus detection in biological materials that have a high host cell background of proteins and nucleic acids. Three milliliters were used for generating the test samples for the HTS study since this volume was used for the inoculation of three cell lines in the in vitro assays per established protocol. The volumes used in the in vivo assays were based on the established protocol for the in vivo assays^1^. Two RNA viruses (single-strand, RSV and double-strand, Reo1) were selected based on their biological properties to ensure at least one virus would produce a positive result in the in vitro and in vivo assays. These viruses were previously detected by HTS in different matrices^15,18^. Six test samples with different spike levels were selected for the study based on an initial pre-study that was conducted to test a range of virus spike dilutions with known virus titer and genome copy number (described below).

### Characterization of viruses and CHO-K1 cell matrix

The RSV and Reo1 viruses that were used for spiking were CBER Next Generation Sequencing Virus Reagents^19^. The Certificate of Analysis (COA) provided the infectious titer and genome copies per milliter (GC/mL) for each virus (details are provided in the Methods). A CHO-K1 cell line producing a recombinant protein (designated as CHO-mAb02) was selected as the background matrix for spiking with viruses to represent potential adventitious virus contamination in an unprocessed harvest material produced during the manufacturing of a CHO-cell-based recombinant protein product. The background material was characterized for protein and nucleic acid content to consider the detection of the spiked viruses in the context of the host cell background in the test sample. Cells and supernatant were harvested from a bioreactor by Lab 3 when the total cell density was 2.05 × 10^7^ cells/mL and the viable cell density was 1.87 × 10^7^ cells/mL (91.3% viability). The titer of the CHO-cell expressed monoclonal antibody was 1.45 mg/mL. The total protein concentration determined by Lab 1 using the Bicinchoninic Acid (BCA) Assay was 4.11 mg/mL and the concentration of CHO host cell proteins (HCP) by ELISA was 0.288 mg/mL. CHO cell DNA was quantified using a ddPCR assay targeted for the Chinese hamster hydroxymethylbilane synthase (HMBS). The result indicated that the genome copy number was 1.13 × 10^6^ GC/mL. Additionally, the testing for mycoplasma, bacteria and fungi demonstrated the absence of microbial agents.

### Pre-study to identify test samples

A series of virus dilutions was spiked into the CHO cell bioreactor material and tested in a pre-study using in vitro infectivity and ddPCR assays, which were performed by Lab 3 and Lab 1, respectively (Table 1). The virus spike levels in the samples were blinded (samples A–N were for testing RSV and samples A–M were for testing Reo1). For the in vitro infectivity studies, the range tested for RSV was 1.08 × 10^4^ to 1.08 × 10^−8^ TCID_50_/mL and for Reo1 1.10 × 10^7^ to 1.10 × 10^−4^ TCID_50_/mL. An abbreviated version of the conventional in vitro cell culture assay for adventitious virus detection was performed in the pre-study, by using two cell lines (Vero and MRC-5) and reading for cytopathic effect (CPE) at day 7 (the conventional assay is described in the Methods). ddPCR assays were done on 10-fold dilutions of the spiked samples containing 1.04 × 10^6^ to 1.04 × 10^−1^ GC/mL of RSV and 1.40 × 10^4^ to 1.40 × 10^0^ GC/mL of Reo1. Unspiked sample was included as negative control for the in vitro and ddPCR assays. The results for the pre-study with RSV and Reo1 are shown in Table 1.

**Table 1:** Pre-study results and samples selected for RSV and Reo1 spiking study

Virus	Sample ID	Viral copy number	Virus infectious titer	Pre-study results		Samples selected for the Study		
		GC/mL	TCID_50_/mL	ddPCR^a^	In vitro^b^	HTS	In vitro	In vivo^c^
RSV	A	Unspiked	Unspiked	-	-	✓	✓	✓
	B	1.04 × 10^7^	1.08 × 10^4^	NT^d^	+	NT	NT	✓
	C	1.04 × 10^6^	1.08 × 10^3^	+	+	✓	✓	NT
	D	1.04 × 10^5^	1.08 × 10^2^	+	-	✓	✓	NT
	E	1.04 × 10^4^	1.08 × 10^1^	+	-	✓	✓	NT
	F	1.04 × 10^3^	1.08 × 10^0^	+	-	✓	✓	NT
	G	1.04 × 10^2^	1.08 × 10^−1^	-	-	✓	✓	NT
	H	1.04 × 10^1^	1.08 × 10^−^^2^	-	-	✓	✓	NT
	I	1.04 × 10^0^	1.08 × 10^−3^	-	-	NT	NT	NT
	J	1.04 × 10^−1^	1.08 × 10^−4^	-	-	NT	NT	NT
	K	1.04 × 10^−2^	1.08 × 10^−5^	NT	-	NT	NT	NT
	L	1.04 × 10^−3^	1.08 × 10^−6^	NT	-	NT	NT	NT
	M	1.04 × 10^−4^	1.08 × 10^−7^	NT	-	NT	NT	NT
	N	1.04 × 10^−5^	1.08 × 10^−8^	NT	-	NT	NT	NT
Reo1	A	Unspiked	Unspiked	-	-	✓	✓	✓
	B	1.40 × 10^7^	1.10 × 10^7^	NT	+	NT	NT	✓
	C	1.40 × 10^6^	1.10 × 10^6^	NT	+	✓	✓	✓
	D	1.40 × 10^5^	1.10 × 10^5^	NT	+	✓	✓	✓
	E	1.40 × 10^4^	1.10 × 10^4^	+	+	✓	✓	✓
	F	1.40 × 10^3^	1.10 × 10^3^	-	+	✓	✓	✓
	G	1.40 × 10^2^	1.10 × 10^2^	-	-	✓	✓	✓
	H	1.40 × 10^1^	1.10 × 10^1^	-	-	✓	✓	✓
	I	1.40 × 10^0^	1.10 × 10^0^	-	-	NT	NT	NT
	J	1.40 × 10^−1^	1.10 × 10^−1^	NT	-	NT	NT	NT
	K	1.40 × 10^−2^	1.10 × 10^−2^	NT	-	NT	NT	NT
	L	1.40 × 10^−3^	1.10 × 10^−3^	NT	-	NT	NT	NT
	M	1.40 × 10^−4^	1.10 × 10^−4^	NT	-	NT	NT	NT

^a^ddPCR assay was done in triplicate. +, positive based on at least 10 droplets; -, less than 10 droplets could not be quantified. For Lab 1, the pre-study samples selected for the main study, were used for HTS.

^b^In vitro, 7-day assay was done for the pre-study (28-day assay was done for the main study). +, positive for CPE; -, no CPE observed.

^c^In vivo, samples used in the main study were based on initial results with the highest spike level.

^d^*NT* Not tested.

✓, Selected for main spiking study.

Interim data were reviewed on unblinded samples by individuals who were not directly involved in handling the samples and obtaining the results. Based on the results shown in Table 1, sample A, and samples C to H were selected as the test samples for both RSV and Reo1 for the head-to-head comparison of HTS and the in vitro assays. For the in vivo assays, RSV testing was carried out with sample B, the highest spiked level containing 1.04 × 10^7^ GC/mL (corresponding to 1.08 × 10^4^ TCID_50_/mL). For Reo1, the samples tested (B–H) contained 1.40 × 10^7^ to 1.40 × 10^1^ GC/mL (corresponding to 1.10 × 10^7^ to 1.1 × 10^1^ TCID_50_/mL). To minimize animal use, the testing with additional spiked levels in the in vivo assays was performed based on initial results obtained with the highest virus dose.

### **Evaluation of virus detection in spiked study samples**

To minimize variability in preparation of the test samples and obtain unbiased study results, single-use spiked samples were prepared by Lab 1 and blinded for distributing to study participants. Each lab used their own HTS workflows for sample extraction, cDNA synthesis, library preparation, sequencing, and bioinformatics pipelines, and used the Reference Virus Database^20,21^ (RVDB) for data analysis. Similarly, each laboratory performed the in vitro assays and interpreted the results according to their established protocols and criteria. The different assays are summarized in Tables 2 and 3, for RSV and Reo1 detection, respectively. The detailed data for the in vitro and in vivo analyses are shown in Tables 5 and 6, respectively, and for the HTS in Supplementary Table S1.

**Table 2:** Results for RSV detection by the different test methods

Sample ID	RSV		HTS^a,b^			In vitro		In vivo^c^
	Viral copy number	Virus infectious titer	Lab 1	Lab 2	Lab 3	Lab 2	Lab 3	Lab 3
	GC/mL	TCID_50_/mL	Targeted/Nontargeted	Targeted/Nontargeted	Targeted/Nontargeted	28-day assay	28-day assay	Primary/Passage
A	Unspiked	Unspiked	- /-	- /-	- /-	-	-	-/-
B	1.04 × 10^7^	1.08 × 10^4^	NT^d^	NT	NT	NT	NT	-/-
C	1.04 × 10^6^	1.08 × 10^3^	+/+	+/+	+/+	+	+	NT
D	1.04 × 10^5^	1.08 × 10^2^	+/+	+/+	+/-	+	+	NT
E	1.04 × 10^4^	1.08 × 10^1^	+/+	+/-	-/-	+	-	NT
F	1.04 × 10^3^	1.08 × 10^0^	+/+	-/-	-/-	+	-	NT
G	1.04 × 10^2^	1.08 × 10^−1^	-/-	-/-	-/-	+	-	NT
H	1.04 × 10^1^	1.08 × 10^−2^	-/-	-/-	-/-	-	-	NT

^a^Positive criteria for targeted analysis was > 2 reads for Lab 1 and ≥ 1 reads for Lab 2 and 3.

^b^Positive criteria for non-targeted analysis for each lab were: Lab 1: positive if 3 or more reads aligned to the reference virus genome were present, and without non RSV or Reo1 taxa; Lab 2: ≥10 reads were observed for a species and ≥ 300 nucleotides were covered on the species accession sequence capturing the highest number of reads; Lab 3: ≥10 reads and coverage breadth ≥25% on the reference virus genome.

^c^RSV was done with the highest spiked level in in vivo assays.

^d^*NT* Not tested.

**Table 3:** Results for Reo1 detection by different testing methods

Sample ID	Reo1		HTS^a,b^			In vitro		In vivo^c^
	Viral copy number	Virus infectious titer	Lab 1	Lab 2	Lab 3	Lab 2	Lab 3	Lab 3
	GC/ mL	TCID_50_/ mL	Targeted/ Nontargeted	Targeted/ Nontargeted	Targeted/ Nontargeted	28-day assay	28-day assay	Primary/ Passage^b^
A	Unspiked	Unspiked	-/-	-/-	-/-	-	-	-/-
B	1.40 × 10^7^	1.10 × 10^7^	NT^d^	NT	NT	NT	NT	+/-
C	1.40 × 10^6^	1.10 × 10^6^	+/+	+/+	+ /-	+	+	+/+^e^
D	1.40 × 10^5^	1.10 × 10^5^	+/+	+/-	-/-	+	+	+/-
E	1.40 × 10^4^	1.10 × 10^4^	+/+	-/-	-/-	+	+	+/ -
F	1.40 × 10^3^	1.10 × 10^3^	-/-	-/-	-/-	+	+	-/ -
G	1.40 × 10^2^	1.10 × 10^2^	-/-	-/-	-/-	+	+	-/-
H	1.40 × 10^1^	1.10 × 10^1^	-/-	-/-	-/-	+	+	-/-

^a^Positive criteria for targeted analysis was > 2 reads for Lab 1 and ≥ 1 read for Labs 2 and 3.

^b^Positive criteria for non-targeted analysis for each lab were as described in Table 2.

^c^Reo1 result was positive based on clinical signs in suckling mice.

^d^*NT* Not tested.

^e^Individual animals subpassaged (except in sample C where organs harvested from individual animals and were then subpassaged).

### **HTS analysis for virus detection**

Different levels of virus detection were seen across the three laboratories. The inter-laboratory sensitivity of detection for RSV ranged from 1.04 × 10^3^ GC/mL to 1.04 × 10^5^ GC/mL. For Reo1, the sensitivity ranged from 1.40 × 10^4^ GC/mL to 1.40 × 10^6^ GC/mL. The overall results are shown in Tables 2 and 3 and the details are provided in the Supplementary Table S1. The difference in the results may reflect some differences in the protocols and highlight the steps that may influence the sensitivity of virus detection by HTS.

Some of the steps in the protocols in the HTS workflow are presented in Table 4 to indicate variables that may contribute tothe different results. It should be noted that the difference in the read number in Lab 1 versus Labs 2 and 3 did not contribute to the sensitivity of virus detection (Tables 2 and 3).

**Table 4:** Differences in some key HTS steps across the laboratories

Protocol Step	Lab 1	Lab 2	Lab 3
Pre-Treatment	Clarification of cells (low-speed centrifugation)	Pre-lysing of cells (freeze-thaw)	Clarification of cells (low-speed centrifugation)
Nucleic acid extraction	Phenol/chloroform/isoamyl	Silica-based column	Silica-based column
Ribosomal RNA depletion	+ (including heat-denature in 5% dimethyl sulfoxide)	+ (RNAs and DNAs were cleaned up and concentrated)	Not used
Sequencer	NextSeq 500/550; 101 cycles for paired-end reads	NovaSeq 6000; 151 cycles for paired-end reads	NovaSeq 6000; 151 cycles for paired-end reads
Samples pooled	No	Yes	Yes
Average number of reads/sample	~10^8^	~10^9^	~10^9^

All the laboratories performed both targeted and non-targeted HTS bioinformatics analyses using their own pipelines and cut-off criteria for determining positive hits. For Lab 1, the targeted analysis showed two mapped reads in the unspiked CHO sample for RSV (Supplementary Table 1). Further non-targeted analysis of the unspiked CHO sample demonstrated the RSV sequences were not of the reference RSV genome but of another RSV type, which had been previously analyzed in the sequencing facility. The unspiked sample for the Reo1 was negative. Therefore, Lab 1 applied the criteria to call the sample as positive when there were more than 2 reads mapping to the virus genome in the sample. The results of the targeted and non-targeted analyses were similar for Lab 1, with results showing that RSV and Reo1 were detected at the 1.04 × 10^3^ GC/mL and at the 1.40 × 10^4^ GC/mL spike levels, respectively (Tables 2 and 3).

In case of Lab 2, no Reo1 or RSV read was observed in unspiked samples following targeted analysis. A sample was therefore called positive if at least one RSV or Reo1 read was found. The results showed that RSV and Reo1 viruses can be detected at 1.04 × 10^4^ GC/mL and 1.40 × 10^5^ GC/mL by targeted analysis, respectively. For non-targeted analysis, Lab 2 showed that RSV and Reo1 were detected at the 1.04 × 10^5^ GC/mL and at the 1.40 × 10^6^ GC/mL spike levels, respectively.

For Lab 3, targeted analysis was conducted for both Reo1 and RSV, defining a sample as positive when any read mapped to the viral reference genome. The result showed that that RSV and Reo1 viruses were detected, respectively, at 1.04 × 10^5^ GC/mL and 1.40 × 10^6^ GC/ml by targeted analysis. For non-targeted analysis, Lab 3 showed that RSVwas detected at the 1.04 × 10^6^ GC/mL spike level and Reo1 was not detected.

The detection levels of the targeted and non-targeted analyses were concordant for Lab 1. However, RSV and Reo1 detection were one log less sensitive with the non-targeted analysis byLab 2 and 3. It should be noted that different criteria (indicated in footnote in Table 2 and detailed in the Methods) were used for determining if a result was positive, particular for non-targeted analysis, which could contribute to the difference in the results.

### In vitro **infectivity analysis**

The set up of the in vitro assay was similar in Lab 2 and 3, (i.e., 28-days with a subpassage at day 14), although there were some differences in the recording of the end-point of the assays (detailed in Materials and Methods) that did not affect the conclusions of the study (shown in Tables 2, 3, and 5).

Briefly, Lab 2 and 3 inoculated three cell lines, i.e., MRC-5, Vero and CHO-K1, with RSV- and Reo1-spiked CHO cell harvest samples, using standard procedures (described in the Methods). The sensitivity of detection for each virus on the selected cell lines was defined as the lowest amount of virus that was detectable in replicate samples by CPE, HA, and HAD or any one of the endpoints in any of the three cell lines. The detailed results obtained by Lab 2 and 3 are presented in Table 5, which indicates the number of wells with positive results for each sample tested in 6 wells (as described in “Methods”).

**Table 5:** In vitro assay data for detection of RSV and Reo1^a^

Spiked virus	Sample ID	Virus titer TCID_50_/mL	Laboratory 2			Laboratory 3		
			Cell lines			Cell lines		
			MRC5	Vero	CHO-K1	MRC5	Vero	CHO-K1
RSV	A	Unspiked	0/6	0/6	0/6	0/6	0/6	0/6
	B	1.08 × 10^4^	NT^b^	NT	NT	NT	NT	NT
	C	1.08 × 10^3^	6/6 D7	6/6 D7	6/6 D14	3/6 D5; 5/6 D10	6/6 D10	0/6
	D	1.08 × 10^2^	4/6 D14	3/6 D7	6/6 D14	1/6 D10; 6/6 D18	6/6 D20	0/6
	E	1.08 × 10^1^	1/6 D7	1/6 D7	6/6 D14	0/6	0/6	0/6
	F	1.08 × 10^0^	6/6 D21	0/6	0/6	0/6	0/6	0/6
	G	1.08 × 10^-1^	6/6 D21	0/6	6/6 D14	0/6	0/6	0/6
	H	1.08 × 10^-2^	0/6	0/6	0/6	0/6	0/6	0/6
	Negative control^c^	NA^e^	0/6	0/6	0/6	0/6	0/6	0/6
Reo1	A	Unspiked	0/6	0/6	0/6	0/6	0/6	0/6
	B	1.10 × 10^7^	NT	NT	NT	NT	NT	NT
	C	1.10 × 10^6^	6/6 D7	6/6 D7	6/6; D14	6/6 D14; 2/2 D28^d^	6/6 D9	6/6 D9
	D	1.10 × 10^5^	6/6 D7	6/6 D7	6/6 D14	6/6 D14; 2/2 D28^d^	6/6 D9	6/6 D9
	E	1.10 × 10^4^	6/6 D7	6/6 D7	6/6 D14	6/6 D28	6/6 D14	6/6 D14
	F	1.10 × 10^3^	6/6 D14	4/6 D7	6/6 D14	6/6 D28	6/6 D14	6/6 D14
	G	1.10 × 10^2^	5/6 D14	3/6 D14	6/6 D14	0/6	6/6 D23	6/6 D28
	H	1.10 × 10^1^	0/6	1/6 D14	6/6 D14	0/6	6/6 D23	0/6
	Negative control^c^	NA^e^	0/6	0/6	0/6	0/6	0/6	0/6

^a^The results of CPE are indicated with number of positive wells/total wells and day (D) of positive result read-out.

^b^*NT* Not tested.

^c^Negative control is medium.

^d^HA with rhesus RBCs at both 2–8 °C and 34–38 °C.

^e^*NA* Not applicable.

In summary, RSV was detected by CPE in at least one indicator cell line by both laboratories (Supplementary Table S2). Lab 2 detected RSV at 1.08 × 10^−1^ TCID_50_/mL in MRC-5 and CHO-K1 cells, and Lab 3 at 1.08 × 10^2^ TCID_50_/mL in MRC-5 and Vero cells. A difference in virus detection was seen between the two laboratories in the three cell lines tested.

The Reo1- spiked harvest was detected by CPE by both laboratories in all three cell lines: Lab 2 detected by CPE with 1.10 × 10^1^ TCID_50_/mL in Vero and CHO-K1 cells and Lab 3 in Vero cells (Table 5). In addition, Lab 3 was able to detect Reo1 in MRC-5 cells using HA with rhesus monkey RBCs at both incubation temperatures (2–8 °C and 34–38 °C) at the two highest titers (1.10 × 10^5^ and 1.10 × 10^6^ TCID_50_/mL) but not at lower spike levels (Supplementary Table 2). Lab 2 did not perform the HA assay since CPE was observed. There was no evidence of detection of HAD on any cell lines with any red blood cell combinations for either RSV or Reo1 in this study. Unspiked sample and media controls results were negative.

### In vivo **results**

Unlike the in vitro tests, which were done by two different labs, the in vivo assays were performed only by Lab 3. Initially, the testing was done on selected dilutions to minimize animal use. The sensitivity of virus detection was defined by the lowest inoculum that resulted in ≥20% mortality or evidence of potential viral infection based on clinical signs. The conventional method defines a failing test as one in which ≥20% of the animals are impacted by the test system and if any animal showed signs of infection by an adventitious agent. Setting the threshold at 20% mortality is a suitable approach, since it is not unusual to lose animals or eggs due to injection trauma, cannibalization, or other microbial contamination relating to the injections. Since RSV detection was negative with sample B in all animal models, other lower dilutions were not considered for testing. Reo1 was negative for adult mice and embryonated eggs at all dilutions. However, for the Reo1 samples, the suckling mice inoculated with samples B to F showed an impact at primary passage related to various clinical symptoms, compared to the animals receiving unspiked sample (Table 6).

**Table 6:** In vivo data for the detection of RSV and Reo1

Spiked virus	Sample ID	Virus titer TCID_50_/mL	Inoculated animals					
			Adult Mice	Embryonated eggs		Suckling mice		
				Allantoic	Yolk sac	Clinical signs		RT-ddPCR
			Surviving/Total	Surviving/Total	Surviving/Total	Primary passage Surviving animals/ Total animals injected	Blind passage Surviving animals/ Total animals injected	Primary passage homogenate
RSV	A	Unspiked	20/20	10/10	10/10	20/20^a^	20/20^a^	-
	B	1.08 × 10^4^	20/20	10/10	10/10	20/20^a^	20/20^a^	-
	C	1.08 × 10^3^	NT^f^	N/A	NT	NT	NT	NT
	D	1.08 × 10^2^	NT	NT	NT	NT	NT	NT
	E	1.08 × 10^1^	NT	NT	NT	NT	NT	NT
	F	1.08 × 10^0^	NT	NT	NT	NT	NT	NT
	G	1.08 × 10^-1^	NT	NT	NT	NT	NT	NT
	H	1.08 × 10^-2^	NT	NT	NT	NT	NT	NT
	Negative control	NA^g^	20/20	10/10	10/10	20/20^a^	20/20^a^	-
Reo1	A	Unspiked	20/20	10/10	10/10	19/20^a^	19/20^a^	-
	B	1.10 × 10^7^	20/20	10/10	10/10	16/20^b^	19/20^a^	+
	C	1.10 × 10^6^	20/20	10/10	10/10	0/20^d^	16/30^c^	NT^e^
	D	1.10 × 10^5^	20/20	10/10	10/10	19/20^c^	20/20^a^	+
	E	1.10 × 10^4^	20/20	10/10	10/10	15/20^c^	20/20^a^	+
	F	1.10 × 10^3^	20/20	10/10	10/10	19/20^c^	19/20^a^	+
	G	1.10 × 10^2^	20/20	10/10	10/10	20/20^a^	20/20^a^	+
	H	1.10 × 10^1^	20/20	10/10	10/10	19/20^a^	19/20^a^	+
	Negative control	NA	20/20	10/10	10/10	20/20^a^	20/20^a^	-

^a^No clinical signs noted in the one animal, which is presented here.

^b^Rough hair coats and enlarged abdomen.

^c^Smaller in size, coat ruffled, swollen abdomen and presence of red/brown fluid when harvested.

^d^Rough hair coats, smaller in size than control animals, sent for necropsy earlier than usual, organs were harvested and passaged into three cages of suckling mice.

^e^All the material was used for the blind passage. The homogenate was not available for RT-ddPCR.

^f^*NT* Not tested.

^g^*NA* Not applicable.

Blind passage was performed for all Reo1 samples using the standard assay protocol and inoculated via the same routes as the primary inoculation. No clinical symptoms were recorded at secondary passage except for Reo1 sample C, and by Day 10 all remaining suckling mice were sent for necropsy and the organs (brain, liver, heart, lung, kidney) were harvested separately from each animal and an organ homogenate was prepared. In order to respect animal use for sample C, only three of the impacted animals were selected and the organ homogenate was injected into litters of 10 suckling mice. By day 9 post injection animals were found dead in each of the passage groups and remaining animals were observed as lethargic with dark fluid in their abdomens. The consistency of clinical signs observed in the suckling mice was influenced by the inherent variability of this animal model: the observation of clinical signs ranged from Day 2–Day 10 in primary passage. All negative control samples that were inoculated in any of the animal models tested did not show clinical signs and the results were negative.

To confirm the in vivo results with RSV and Reo1 infection, RT-ddPCR analyses were performed by Lab 1 using RNAs extracted from in vivo homogenates from suckling mice that were used for the blind passage. The results for both viruses were negative in the in vivo assays for the control samples injected with the matrix only. Detection of RSV RNA was negative and Reo1 RNA was detected in the pooled homogenates of the suckling mice injected at the spike levels that were tested, i.e., samples B, and D to H. These results suggest the clinical signs observed in the Reo1 virus-injected suckling mice were due to virus infection and that it is also possible to have virus infection in the absence of clinical signs, as shown with Reo1 spikes G and H.

## Discussion

This study compared HTS with the currently recommended conventional in vitro cell culture and in vivo animal assays for sensitivity of adventitious virus detection in a complex biological mixture. The test material used for spiking with the two model viruses was unprocessed (unpurified) harvest from a CHO-K1 cell line producing a monoclonal antibody and grown in a bioreactor to represent material that may be tested during product manufacturing, i.e. containing whole or disrupted cells, and cell-derived nucleic acids and proteins, in addition to a recombinant antibody. To our knowledge, this is the first study to perform a head-to-head comparison of virus detection using the same test samples in the in vitro assays, in vivo assays, and HTS. RSV and Reo1 were selected based on the knowledge of their susceptibility for virus infection in at least one of the targets in the in vitro and in vivo assays^22–24^. The selection of the two viruses was not intended to show the breadth of HTS, as this has already been demonstrated^15,18,25,26^, but to assess the analytical sensitivity of the three methods for their suitable application for viral testing of biologics.

In the next sections, each method will be discussed in detail, allowing to highlight key differences between the methods that could impact the sensitivity of the detection of Reo1 and RSV in the unprocessed harvest matrix.

### In vitro **assays**

The conventional in vitro cell-culture virus detection assays are currently recommended for product safety by testing starting materials including virus seeds and cell substrates, and intermediate materials produced during biological product manufacturing and used for generating the final product. Generally, the in vitro testing requires three different cell lines, and the results are reported based on a combination of CPE, HAD, and HA endpoints analysis^1,3^. In the present study, the spiked viruses were chosen based on their ability to replicate in at least one of the three target cell lines^24^ and produce a biological effect. Although the detection of the two model viruses was expected, the sensitivity of detection in the conventional assays and HTS was not known for determining the suitability of HTS as an alternative method for replacing the current assays for adventitious virus detection.

The present study indicated some of the limitations of the in vitro assays. In this study, RSV was detected by CPE only, while Reo1 was also detected by HA, using rhesus monkey RBCs at both 2–8 °C and 34–38 °C. HAD was not observed for either virus. It should be noted that not all Reo1 strains produced CPE but do display hemagglutinating properties^27,28^. The results highlight that in vitro assays require using a combination of different cell lines and endpoint assays and virus detection is based on the susceptibility of the target cell for virus infection and replication and the properties of the virus to produce a visible effect in the read-out assays (CPE, HAD or HA). Furthermore, variability in virus detection was seen in the same target cell lines between the two labs using similar protocols. The difference in the results could be due to the source of the cell lines and biological raw materials used for cell growth (e.g., serum). The results demonstrate that the cell line is a critical variable in the assay. The assay variability demonstrates the need to supplement or replace this assay with other analytical methods for broad adventitious virus detection such as PCR virus panels or HTS ^8^. These nucleic acid-based assays could also be used as investigational tools to follow up a positive signal in the cell-based in vitro assay.

It should be noted that the sensitivity of the in vitro assay was improved in both laboratories when the assay was performed for a 28-day period, which included a blind passage at 14 days. This has been reported in previous publications^24^ and is recommended in international guidelines^1,3,8^. The ability to detect virus at 14 days was also shown in this study, however only the higher virus concentrations were detected at this time point, in particular for Reo1, but the sensitivity was improved with 28-day culture.

### In vivo **assays**

The in vivo animal assays are used as a broad-spectrum conventional method to examine detection of viral agents in biologics, although it is recognized that animal studies may not be as sensitive or broadly susceptible for virus detection as compared to in vitro assays^24,29^. In this study the use of animal testing for viral safety of biologics was examined in a head-to-head comparison with in vitro cell culture assay and HTS. This study confirmed the limitations expected for using the in vivo assays. Only Reo1 was detected by the in vivo method while both Reo1 and RSV were detected by in vitro and HTS methods. Furthermore, neither virus was detected in adult mice and embryonated eggs, and RSV was not detected in suckling mice ^30^. However, Reo1 was detected in suckling mice in the primary passage based on clinical signs (samples B to F – 1.10 × 10^7^ to 1.10 × 10^3^ TCID_50_/mL) whereas the subpassage for virus transmission was negative, except in one case (sample C at 1.10 × 10^6^ TCID_50_/mL), which may be due to protocol modification (see Methods). It should be noted that in this study the analysis for virus detection extended beyond the standard protocol where all the results (except sample C) would have been reported as negative since they would be based on the clinical outcome in the subpassages and warranted further investigation due to clinical signs at the primary passage by repeating the study. Furthermore, in vivo results could be verified using ddPCR since the virus in the test sample was known, which is also not standard practice.

The results of the study showed that in vivo assays for virus detection are of limited value due to lack of sensitivity for broad virus detection and the difficulty of accurately interpreting results, while HTS and in vitro assays can detect viruses more broadly with a defined sensitivity.

### HTS

In this study, HTS-targeted bioinformatics analysis was performed to evaluate the sensitivity of detection of the known spiked viruses in a complex matrix and non-targeted bioinformatics analysis was performed to demonstrate virus detection by the pipelines used for broad virus detection, albeit only two viruses were used in this study. Unlike the in vitro cell culture assays and the in vivo animal assays, HTS can detect all nucleic acids in the sample without prior viral sequence knowledge and does not depend on virus replication and other virus biological properties, which are needed for virus detection in the conventional assays. Therefore, non-targeted HTS analysis is recognized as a potential method for replacing the in vitro and in vivo adventitious virus detection assays^8^. However, it is noted that a signal detected by HTS will need further follow up to determine whether it is associated with an intact virus.

In this study, independent targeted and non-targeted bioinformatics pipelines were used by the three labs. Both RSV and Reo1 were detected in a matrix with high cellular background by all the participants. However, the sensitivity of virus detection between the labs varied up to two logs (RSV: 1.04 × 10^5^ to 1.04 × 10^3^ GC/mL; Reo1: 1.40 × 10^6^ to 1.40 × 10^4^ GC/mL). Furthermore, the virus detection using targeted versus non-targeted varied up to one log. These differences in the results between the labs could be due to different sample processing protocols and lab-specific bioinformatics pipeline with dedicated cutoff criteria for a positive result. In this study, a complex matrix was used with a high content of host cell line nucleic acids and proteins, which could impact the sample preparation and processing, as well as downstream bioinformatics analysis. Depending on the lab, freeze-thaw, proteases or low speed centrifugation for clarification of cells were applied as pre-treatment (see Methods). For the RNA extraction, the ribosomal RNA (rRNA) removal seemed to be critical to enhance the sensitivity of virus detection. This step was not performed by Lab 3, which showed the lowest sensitivity of virus detection, while Labs 1 and 2 included that step in their protocol. Regarding the difference between Lab 1 and Lab 2, this may be due to clarification of the cell debris from supernatant during sample preparation. The bioinformatics pipeline may also impact the results for virus detection. For example, removal of CHO genome sequences was done differently between the labs. While Lab 1 removed ribosomal RNA sequences in addition to CHO genome, Labs 2 and 3 filtered only the CHO genome sequences. In addition, Lab 2 combined the host and virus databases into a single virus screening process for host removal and virus detection.

In this study, the two RNA viruses were selected based on their sensitive detection in the in vitro and in vivo assays and to challenge the HTS workflow for detection of double-stranded RNA viruses such as Reo1. The detection of DNA viruses in a high cellular background matrix was not included in this study, although both RNA and DNA viruses were detected in our previous study by spiking in a high titer virus background^15,25,31^.

### **Comparison of HTS**, in vitro and in vivo **assays for virus detection**

The study results indicated a greater sensitivity for RSV and Reo1 detection using the in vitro assays and HTS compared to the in vivo assays. An impact of the complex matrix with a high cellular background was seen with HTS; this is expected for methods based on nucleic acid detection, whereas this did not seem to affect the biological assays with the selected viruses used in this study. Noting this limitation, HTS assay still can provide a greater breadth of virus detection, which was not specifically addressed in this study since it has been shown in other studies and is now generally recognized^15,18,26^. Furthermore, HTS results could be obtained in weeks whereas the results from the in vitro and in vivo assays results were obtained in a month or more.

The difference in sensitivity of virus detection by the different labs indicates that further optimization of HTS is needed to enhance the sensitivity of the method using different types of test materials. This can include analyzing each step of the process such as the sample pre-treatment (matrix dependent), extraction type, rRNA removal, by using viral internal control and reference standards^25,31^ to evaluate the entire HTS workflow from upstream sample processing through downstream bioinformatics. The outcome of such efforts can facilitate development of common standardized processes (SOPs), to establish the technology by defining relevant validity criteria. It is recognized that a positive HTS signal will need further follow up to determine the presence of an infectious virus, which may include using cell culture and other molecular assays.

## Methods

### Viruses

Human respiratory syncytial virus strain A2 (RSV) and mammalian orthoreovirus type 1 strain Lang (Reo1) used in the study were CBER Next Generation Sequencing Reagents (previously designated as WHO international reference reagents for adventitious virus detection in biologics by HTS)^25^. Infectious titer and genome copy number (including particle-associated genomes and free RNA) were provided in the COA for the viruses. This was determined using primers in the N gene for the RSV and in the L2 gene for the Reo1. This information along with the virus properties are shown Table 7.

**Table 7:** Physicochemical and genome characteristics of RSV and Reo1 viruses

Virus Characteristics	Virus and Genome Properties	
	RSV	Reo1
Envelope	Yes	No
Chemical resistance	Low-medium	Medium-high
Genome type	Single-strand RNA, negative, linear, poly A+ tail	Double-strand RNA, segmented, linear, no poly A+ tail
Genome size	15 kb	10 segments: 1,196-3915 nt
Particle size	150–200 nm	80 nm
Genome copy number per mL	1.04 × 10^9^	1.40 × 10^10^
Infectious titer (TCID_50_) per mL	1.08 × 10^6^	1.10 × 10^10^

### Matrix preparation, storage, characterization, and testing

The matrix for virus spiking was provided by Lab 3. CHO-K1 cells (mAb02, MilliporeSigma) producing a recombinant protein were grown in the 200 L Mobius CellReady bioreactor (200 L Mobius Pilot scale, MilliporeSigma) and material collected aseptically was aliquoted into 2 × 2 L sterile PETG bottles in a biosafety cabinet and frozen at -80 °C until ready for use. The material was shipped to Lab 1 for preparation of the spiked samples. Frozen matrix bottles were thawed at 4 °C, combined into a 5 L sterile flask in a biosafety cabinet and stirred slowly with a sterile magnetic stir bar to get a homogenous mixture. Individual matrix aliquots were prepared and stored at -80°C until ready for use. Each aliquot contained the appropriate volume to prepare the different virus spike levels for one-time use in the test assays.

Characterization of the matrix was done by Lab 1 using following assays. Total protein content was quantified by Pierce™ BCA Protein Assay Kit (Cat # 23225, Thermo Fisher Scientific). The clarified supernatant of CHO matrix was exchanged from medium to PBS using Nanosep Centrifugal Devices with Omega™ Membrane 30 K (Cat # OD030C33, Pall Corporation) for concentrating protein and removing phenol red color, which can affect the read-out. The samples were assayed in triplicate according to the manufacturer’s protocol for the microplate procedure. Absorbance was measured at 562 nm using BioTek Synergy 4 microplate reader.

Host cell proteins were characterized using CHO HCP ELISA kit (Cat # F015, Cygnus Technologies). Serial diluted samples were made in triplicate and assayed according to the manufacturer’s protocol. The average of three independent assays were reported. Absorbance was measured at 405/492 nm using BioTek Synergy 4 microplate reader.

Host genome copy number was determined using the ddPCR assay described below.

### Sterility and mycoplasma testing

Both sterility and mycoplasma testing were done to provide assurance of the suitability of the matrix preparation for future studies. The tests were done by Lab 3, which used established pharmacopeial assays in accordance with global regulatory guidance^2,32–37^. Sterility method suitability/qualification studies were performed using 1.5 mL per organism to establish the ability to detect microbial and fungal agents. For the sterility test, the Reo1 harvest was inoculated into two media types (Fluid Thioglycollate media (THIO) and tryptone soya broth (TSB); Millipore BD, STBMFTM12 and STBMTSB12) and further spiked with 100 colony-forming units of specific bacterial species [*Staphylococcus aureus* (ATCC 6538), *Pseudomonas aeruginosa* (ATCC 9027), *Clostridium sporogenes* (ATCC 19404), *Bacillus subtilis* (ATCC 6633), *Candida albicans* (ATCC 10231), *Aspergillus brasiliensis* (ATCC 16404)]. The THIO broths containing test matrix and *S. aureus*, *Ps. aeruginosa*, and *Cl. sporogenes* were incubated at 32 ± 2°C for a maximum of 5 days. The TSB broths containing test matrix and *B. subtilis*, *C. albicans* and *A. brasiliensis* were incubated at 22 ± 2°C for a maximum of 5 days. The broth containers were observed for any turbidity representing microbial growth. Once acceptable results had been achieved the standard sterility assay using a representative volume of material was examined for each virus harvest using the same media and incubation conditions as described above. The test broths were held for a period of 14 days and observed at regular intervals during the test period.

Mycoplasma detection was performed using mycoplasma qPCR technology. The test system employed automated DNA extraction and tested on an automated PCR coupled with BioRad CFX real time technology to detect the presence of mycoplasma species. Test matrix from 5 × 10^5^ cells/mL was extracted using QIAsymphony Certal Vaccine NA kit and coupled with automated QIAsymphony technology. Primers and probes derived from the 16S rRNA region of the Mollicutes phylum were optimized for use to detect mycoplasma species. For the mycoplasma PCR assay, a total of 12 µL of both the RSV and Reo1 harvest material was tested. The PCR assay is qualitative, a Cq value below a cut-off threshold is indicative of the presence of mycoplasma DNA. Relevant controls are included in the system including contamination control, no template control, positive and negative control, internal extraction control and spike control (a duplicate 1 mL aliquot of test article is spiked with 10 cfu of *Mycoplasma orale*). This validates the performance of the test in the presence of test article by confirming that the assay detection limit of 10 cfu/mL is achieved.

### Preparation of virus-spiked material

For virus-spiked sample preparations, RSV or Reo1 was thawed in a 37 °C water bath and 10-fold serial dilutions were prepared in CD FortiCHO media (Cat # A11483-01, Gibco). The subsequent 10-fold dilutions were made in the matrix. Final virus spiked levels were: 1.08 × 10^4^–1.08 × 10^-8^ TCID_50_/mL and 1.04 × 10^7^–1.04 × 10^−5^ GC/mL for RSV, and 1.10 × 10^7^–1.10 × 10^-4^ TCID_50_/mL and 1.40 × 10^7^–1.40 × 10^−4^ GC/mL for Reo1. The negative control, unspiked matrix was prepared by making a 10-fold dilution with the CD FortiCHO media. Single-use aliquots of the spiked and unspiked material were coded to blind the virus concentration from the study participants and frozen at −80 °C before distribution. To avoid cross-contamination, RSV or Reo1 spiked samples were prepared on separate days, and the unspiked matrix was prepared in separate biosafety cabinets by different operators. Single-use aliquots of the spiked materials were made to avoid loss of virus infectious titer due to freeze-thaw and their volumes varied depending on the assay. Samples were shipped for testing to Lab 2 and Lab 3.

### In vitro assays

In vitro testing for adventitious agents was performed in compliance with global guidelines, including European Pharmacopoeia 2.6.16^3^, ICH Q5A(R2)^8^ and 2010 U.S. Food and Drug Administration (FDA) Guidance for Industry^1^. To assess the ability of standard adventitious virus assays to detect potential viral contaminants, serial dilutions of each virus stock in a background of CHO-K1 unpurified bulk harvest material were inoculated onto MRC-5, CHO-K1 and Vero cells grown in 6-well plates.

Lab 2 performed in vitro assays based on their protocol. Following adsorption for 60 min, cells were fed with MEM (GIBCO, 2380238) containing 1% FBS (MOREGATE, 55301118) and neomycin/gentamicin (in house/GIBCO, respectively D22R035912 and 24522430A), and incubated in a humidify 5 ± 2% CO_2_ incubator at 36 ± 1 °C. Growth medium was replenished after 7 days. Monolayers were observed for CPE at day 7 and 14. All positives were stopped and discarded. If no CPE observed at day 14, for Vero (Lab 2 Repository) and MCR-5 (Lab 2 Repository), 0.5 mL of supernatant was reinoculated on fresh set of cells. For CHO-K1 (Lab 2 Repository), culture media from all wells were centrifuged at 200 × *g* (ALLEGRA X-12R) for 10 min at 2–8 °C, and 0.5 mL of supernatant was reinoculated on fresh set of cells. Cells were incubated for additional 14 days. Growth medium was replenished on day 21. Monolayers were observed for CPE at days 21 and 28. All positives were stopped and discarded. If no CPE observed at day 28, cells were removed from the incubator and tested for hemagglutination (HA) or hemadsorption (HAD) of red blood cells (RBCs). Briefly, in cases where HA was assessed, an aliquot of supernatant was removed, centrifuged at 200 × *g* for 10 min at 2-8°C, and tested for haemagglutination using guinea pig (Lab 2 in-house repository) erythrocytes following incubation at 2–8 °C and 36 ± 2°C. In addition, cells from day 28 in all wells were incubated with guinea pig erythrocytes at 2–8 °C and 20–25 °C for 30 min each. In cases where HAD was assessed, cell monolayers were overlaid with a suspension of guinea pig erythrocytes. The plates were observed for adsorption of the RBCs to the cell monolayers after sequential 30-min incubations at 2–8 °C and 20–25 °C. Scoring for each virus on each indicator cell line (MRC-5, Vero, and CHO-K1) was defined as positive by detection of virus based on any of the endpoints used.

Lab 3 performed in vitro assays based on their protocol. Following adsorption for 60–80 min, the cells were fed in either Eagle’s modified essential medium (EMEM) (Cat # M4655-500ML, Sigma) for MRC-5 and Vero cells or Ham’s F12 (Cat # 11765-054, GIBCO) for CHO-K1 cells. All media were supplemented with 2% FBS (Cat # F4135, Sigma) and gentamicin and amphotericin B (Cat # 015-10, GIBCO) and incubated in humidified 5 ± 2% CO2 incubators at 34–38 °C. Monolayers were observed for CPE at regular intervals for 14 days. Growth medium was replenished as required. On day 14, cells were removed from the incubator and tested for hemagglutination (HA) or hemadsorption (HAD) of red blood cells (RBCs). Briefly, in cases where HA was assessed, an aliquot of growth medium was removed and tested for hemagglutination by incubation with a suspension of chicken (Cat # 7241408, Lampire), guinea pig (Cat # 7243108, Lampire), or rhesus monkey erythrocytes (Cat # 7248508, Lampire) at 2–8 °C and 34–38 °C. In cases where HAD was assessed, duplicate wells were overlaid with a suspension of chicken, guinea pig, or rhesus monkey erythrocytes. The plates were observed for adsorption of the RBCs to the cell monolayers after sequential 30 min incubations at 2–8 °C and 34–38 °C. A blind passage of the growth medium from cells was performed for negative control and all virus seed samples that appeared to be morphologically normal i.e., without CPE, on day 14. These monolayers were observed for CPE at regular intervals for an additional 14 days. Growth medium was replenished as required. On day 28, cells were removed from the incubator and tested for HA or HAD using RBCs as described above. Concurrent positive controls (MRC-5/measles virus (Edmonton strain; ATCC VR-24), Vero/bovine parainfluenza virus (ATCC VR-281) and CHO-K1/simian virus type 5 (ATCC VR-288) and negative controls (EMEM + 2% FBS) were performed during the experimental phase of the study and a valid test was confirmed by recovery of virus in each cell line and no CPE/HA/HAD observed in the negative control cultures. Scoring for each virus on each indicator cell line (MRC-5, Vero, and CHO) was defined as positive by detection of virus on any of the endpoints used.

### In vivo a**ssays**

Lab 3 performed the in vivo assays based on their protocol. In order to minimize animal use, the in vivo testing was done one time at pre-selected dilutions and performed at only testing facility (Lab 3). All animals were maintained in accordance with the *Guide for the Care and Use of Laboratory Animals* under an approved protocol by the Institute Animal Care and Use Committee. For the suckling mice, all routes of injection are performed without anesthesia due to their age. For the adult mice, all routes of injection are performed under anesthesia. The anesthesia is administered with a vaporizer in a chamber containing a mixture of isoflurane and oxygen. Euthanasia is performed using CO2 using a Euthanex machine. In vivo testing for adventitious agents was performed in compliance with U.S. regulatory methods^1^. The in vivo assays were performed using suckling mice, adult mice, and embryonated hens’ eggs. Animals were obtained from Envigo (Frederick, MD, U.S.A.) and SPF eggs from Charles River (Norwich, CT, U.S.A.). To minimize cross-contamination, all open manipulations are performed in a Class II Biological Safety Cabinet, and animals were housed in static filter-topped Microisolator cages in a negative-pressure work area.

The test sample was received frozen and thawed prior to inoculation. Suckling mice (1–4 days old, two litters with 10 suckling mice per litter) were inoculated by the intraperitoneal (0.1 mL), intracerebral (0.01 mL) and per os (0.01 mL) routes and adult mice (at least 5 weeks old, 10 adult mice of 5 male and 5 female mice) were inoculated by the intraperitoneal (0.05 mL), intracerebral (0.03 mL), per os (0.05 mL) and intranasal (0.05 mL) routes. Embryonated eggs were inoculated with 0.1 mL of the test matrix into the allantoic cavity (10–11 day old embryonated hens’ eggs, 10 eggs per test sample) and a further 0.1 mL into the yolk sac (6–7 day old embryonated eggs, 10 eggs per test sample). The mice or eggs were observed for mouse mortality or death of the embryo. Negative control animals (same number of adult mice, suckling and embryonated eggs as test sample) were included in the test. Negative control samples were HBSS inoculated in embryonated eggs and mice via the same routes of inoculation and using the same volumes as the test animals.

The test analysis is considered valid when at least 80% of the negative control and test matrix samples in embryonated eggs, adult mice and suckling mice survive the 28-day test period in good health including those eggs and animals, which succumb to loss due to accident or trauma. From the primary passage, samples from surviving embryos or suckling mice are passaged into naïve embryonated eggs or suckling mice according to standard procedures. In the suckling mice portion of the assay, pooled sample homogenate from euthanized mice was inoculated in an additional group of mice and observed for a further 14 days or earlier if the animals were deemed impacted by the test sample. Alternatively, a homogenate of the pooled organs (heart, lung, liver, brain and kidney) may also be prepared from suckling mice and used to also inoculate a group of suckling mice with further observation for an additional 14 days. Negative controls were included and injected with HBSS.

Allantoic fluid is further examined at both the primary and passage of the test system. The fluids are harvested and assayed for HA using chicken, guinea pig and Human O erythrocytes. Fluids are observed for HA activity after incubation for 1–2 h at 2–8 °C and 21–25 °C after 1–2 h at ambient temperature. The results of the HA are interpreted based on successful results from both the negative and positive controls. HA levels above background must not be observed in allantoic fluids from the first of second passage eggs inoculated with the text matrix via the allantoic route. Clinical signs were recorded throughout the test process at both the primary and secondary passage stages of the test.

### Nucleic acid extraction of homogenates from in vivo primary inoculation of suckling mice

For the analysis of the presence of RSV or Reo1 infection in the suckling mice, homogenates were generated from the in vivo assays. Frozen vials of the homogenates (prepared 14-days post-injection) were received by Lab 1 for RNA extraction and RT-ddPCR analysis using the RSV and Reo1 primers shown in Table 8. For RSV, samples A and B were provided (unspiked and 1.08 × 10^4^ TCID_50_/mL/1.04 × 10^7^ GC/mL) and for Reo1 sample A, B, D-H were provided (unspiked; 1.10 × 10^7^ TCID_50_/mL/1.40 × 10^7^ GC/mL; 1.10 × 10^5^ - 1.10 × 10^1^ TCID_50_/mL/1.40 × 10^5^ - 1.40 × 10^1^ GC/mL, respectively). Total RNA was extracted from pools of organs from mice in 2 cages with 6–10 suckling mice per cage at the time of sample collection. 100 µl of each homogenate was extracted using the RNeasy Plus Universal kit (Cat # 73404, Qiagen) according to the manufacturer’s protocol. The RNA was eluted in 100 µl of nuclease-free water and quantified with the Qubit RNA Broad Range (BR) kit (Cat # Q10210, Invitrogen). 100 ng of the RNA was used to detect the presence of the virus by ddPCR as described above.

**Table 8:** ddPCR primers and probes

Primer/Probe	5’ to 3’ sequence	bp
RSV/N_Forward	GCAAATATGGAAACATACGTGAACA	25
RSV/N_Reverse	GCACCCATATTGTAAGTGATGCA	23
RSV/N_Probe	FAM- CTTCACGAAGGCTCCACATACACAGCTG -BHQ1	28
Reo1/L2_Forward	TGATGCTAACAAAGGAGAGTGG	22
Reo1/L2_Reverse	AAACGAACCCAGAGACACAG	20
Reo1/L2_Probe	FAM- AGCGTCTGAGAACACCATGTCCAAT -BHQ1	25
CHO HMBS_ Forward	CTCAGTTGCTATGTCTACCAC	21
CHO HMBS_ Reverse	CGTATTCTAGCTCCTTGGTAAAC	23
CHO HMBS_ Probe	FAM- TGATACTGCACTCTCTAAGGTAATGACAGC -BHQ1	30

### HTS

For Lab 1, to conserve the limited number of test sample aliquots, nucleic acids extracted for ddPCR (described above) were also used for HTS analysis.

Samples were thawed in a 37 °C water bath with gently swirling followed by low-speed centrifugation (Allegra 6KR, swing bucket rotor, Beckman coulter) at 1200 × *g* for 10 min at 4 °C to remove cells and debris. The clarified supernatant (3.0 mL) was removed for nucleic acid extraction using phenol: chloroform: isoamyl alcohol (PCI).

Nucleic acid was extracted from 3.0 mL sample by adding 1X lysis buffer [10 mM Tris-HCl, pH 7.5 (Cat # 15567-027, Invitrogen), 125 mM NaCl (Cat # AM9759, Invitrogen) and 0.625% SDS (Cat # V6551, Promega) in the final concentration] with 1 mg/mL of proteinase K (Cat # 03115828001, Roche) and incubating for 3 h at 56 °C. To the mixture was added equal volume of phenol: chloroform: isoamyl alcohol (25:24:1, v/v, Cat # 15593031, Invitrogen) with high-speed vortexing of 30 s. After spinning at 6500 rpm for 10 min at room temperature, the aqueous phase was transferred to a 50 mL conical tube (Cat # 430828, Corning), added 2.5 volume of ice-cold absolute ethanol (Cat # E7023, Sigma-Aldrich), and the sample was mixed by inverting and stored at −20 °C overnight. The sample was then centrifuged at 6500 rpm for 1 h at 4 °C. After discarding supernatant, pellet was washed with 70% ice-cold ethanol 2 times with quick vortex before centrifuging at 6500 rpm for 30 min at 4 °C. The pellet was air-dried for 15 min, resuspended in 600 µL of nuclease-free water (Cat # 351029721, Quality Biological) and aliquots were made for storage at −80 °C. Total RNA concentration was measured by Qubit 3 fluorometer (Thermal Fisher, USA) using the RNA broad range assay kit (Cat # Q10211, Invitrogen) and the quality was examined by the NanoDrop ND-1000 spectrophotometer (Thermal Fisher, USA).

cDNA synthesis and library preparation were done using the TruSeq® Stranded Total RNA Library Prep Gold (Cat # 20020599, Illumina) according to the manufacturer’s instructions, with TruSeq RNA Single Indexes Set A (Cat # 20020492, Illumina). The following modification was included in the library preparation: after pulldown-based rRNA depletion from 1 μg of RNA, RNA fragmentation was done by 8 min at 94 °C with 5% DMSO for both RSV and Reo1 spiked samples. The evaluation of DNA fragment size was applied by Agilent 2100 bioanalyzer with DNA 1000 reagent (Cat # Q10211, Agilent Technologies) and library quantification was performed using the Qubit dsDNA broad range assay kit (Cat # Q32853, Invitrogen). The sequencing libraries were loaded onto NextSeq platform with 500/550 Mid Output Kit v2.5 (300 cycles) (Cat # 20024905, Illumina) and paired-end reads of 2 × 101 cycles was performed. Each sample was sequenced individually, and index read was not included in the run. A denatured PhiX v3 library (Cat # 5067-1504 Illumina) was spiked 1% (v/v) into the library prior to sequencing as the internal control.

For targeted analysis, the sequencing raw reads were converted to FASTQ files by bcl2fastq program and processed by CLC Genomic Workbench version 22.0.2 (Qiagen). Unless mentioned, the modules were applied with the default settings. The raw reads were trimmed to remove the sequencing adapter and low-quality reads by *Trim Reads* module with the adapter list provided by Illumina. The trimmed reads were mapped to RSV (GenBank: JF920069.1), Reo1 (GenBank: M24734.1, AF378003.1, AF129820.1, AF461682.1, AF490617.1, AF174382.1, M10260.1, L19774.1, M18389.1, and X61586.1) CHO genome (GenBank: GCF_000223135.1_CriGri_1.0_genomic_tran), in-house CHO rRNA collection (Supplemental Table 3) and PhiX internal control (NC_001422.1) by using *Map Reads to Reference* module. The mappings for RSV and Reo1 were performed with the high stringency parameters (Length = 80, Similarity = 90) to avoid any false-positive calls while other mappings were remained as default. The mapped tracks were subjected to *QC for Read Mapping* module to obtain the information of genome coverage and sequencing depth. The reads indicating the positive calls were extracted and subjected to NCBI BLASTN alignment^38^ against nt. The detection of RSV or Reo1 was called positive if more than two aligned reads were present, and the aligned reads had no non-viral taxa screened by BLASTN against NCBI nr/nt database.

For non-targeted analysis, the adventitious virus detection was performed by an in-house non-targeted detection pipeline. The default parameters of tools were used unless indicated. Briefly, the raw FASTQ reads were trimmed by BBtools BBDuk v38.69^39^ to remove the adapter, low quality, and low complexity reads, and the adapter list provided by Illumina was imported to the trimmer. The quality matrix of reads before and after trimming was evaluated by FastQC v0.11.8^40^. The host contents were removed from the quality-trimmed reads by HISAT2 v2.2.1^41^ by using CHO genome (GenBank: GCF_000223135.1_CriGri_1.0_genomic_tran) and in-house CHO rRNA (Supplemental Table 3) as the indexes. The host-unmapped reads were extracted by SAMtools v1.12, and the paired-end reads were merged by BBTools v38.69^39^. The merged reads were subjected to CD-HIT-EST v4.8.1^42^ to remove the redundant reads sharing 100% similarity. The de-duplicated reads were subjected to BLASTN v2.10.1+ alignment against U-RVDB v24.1 to identify the viral signals. The reference genomes of candidates were extracted, indexed and subjected to HISAT2 v2.2.1^41^ along with the quality-trimmed, non-collapsed reads for the realignment process.

Counter screening was performed to verify the fidelity of viral signals: the QC-trimmed reads resulted in the potential calls from HISAT2 realignment were extracted and subjected to BBtools BBDuk v38.69 to remove the low complexity reads. The processed reads were further subjected to BLASTN v2.10.1+ analysis against nt (database downloaded on Nov. 16, 2022). The candidates were confirmed to be truly positive calls if the reads of such candidate were exclusively related to the designated taxonomy revealed by the realignment process with no crosstalk other than viral taxonomies. The final candidate lists were generated as tab-delimited format (tsv) and imported to Microsoft Excel 2021 for further review and analysis. The detection of RSV or Reo1 was called positive if 3 or more aligned reads were presented, and without non RSV or Reo1 taxa.

Lab 2 initially aliquoted samples at 200 µL in 2 mL Eppendorf tubes. Each aliquot was pre-lysed using the freeze/thaw method: three times 5 min in Absolute ethanol/carbon ice and 5 min at 37 °C.

Total nucleic acids (DNA and RNA) were extracted using the QIAamp MinElute Virus Spin Kit (Cat # 57704, Qiagen). Extracted nucleic acids were cleaned and concentrated using the RNeasy MinElute CleanUp Kit (Cat # 74204, Qiagen; 5 × 200 µL per conditions).

The RNA concentration was evaluated using the Qubit RNA Broad Range (BR) kit (Cat # Q10210, Invitrogen) on a Qubit fluorometer (Invitrogen).

It was followed by a ribosomal RNA (rRNA) removal using RiboMinus Eukaryote System v2 kit (Cat # A15026, Invitrogen; 5 µg per tube).

The double-stranded complementary DNA (cDNA) synthesis was performed using the SuperScript IV Reverse Transcriptase (Cat # 18090050, Invitrogen) and the Second Strand cDNA Synthesis Kit (Cat # A48570, Invitrogen).

The cDNA aliquots were pooled, cleaned and concentrated using the MinElute PCR Purification Kit (Cat # 28004, Qiagen).

cDNAs were quantified using Qubit dsDNA High Sensitivity (HS) kit (Cat # Q32850, Invitrogen) on a Qubit fluorometer (Invitrogen).

Sequencing libraries were prepared from cDNA by tagmentation using Illumina DNA Prep kit (Cat # 20018704, Illumina; 1 ng of cDNA per sample) and indexes added using the IDT for Illumina DNA/RNA UD Indexes Set A, Tagmentation (Cat # 20027213, Illumina; 96 Indexes, 96 Samples) according to the manufacturer’s instructions. RNA-Seq analysis was done in one run on an Illumina NovaSeq 6000 instrument using the NovaSeq 6000 S4 Reagent Kit v1.5 (Cat # 20028312, Illumina; 300 cycles). The sequencing was performed using the following parameters: 2 × 151 cycles paired-end sequencing (according to Illumina’s instructions), pooled library at 2.25 nM each, PhiX control v3 (Cat # FC-110-3001, Illumina) at 250 pM.

Raw sequencing reads were first demultiplexed using bcl-convert v3.10.5 to extract all reads pertaining to each sample. Quality of paired-end sequencing reads was first checked using the FastQC v0.11.9 software (https://www.bioinformatics.babraham.ac.uk/projects/fastqc).

For each sample, trimming of adapter/index sequences and trimming of low-quality bases was achieved using Trimmomatic v0.39^43^ with a minimum read length of 50 bp (options -phred33 ILLUMINACLIP: TruSeq3-PE.fa:2:30:10 SLIDINGWINDOW:4:20 LEADING:3 TRAILING:3 MINLEN:50). After this stage, all remaining reads were considered high-quality reads and ready to be analyzed.

For targeted analysis, high-quality reads associated to each sample were processed using a Lab 2 internal data analysis pipeline for targeted adventitious virus detection. High-quality reads were aligned using BWA v0.7.17^44^ (options mem -M -a) to a custom sequence database containing NCBI reference sequences for CHO, RSV, and Reo1: *Cricetulus griseus* (Chinese hamster, CHO, GenBank: GCF_003668045.3), Human orthopneumovirus (RSV, GenBank: JF920069.1), Mammalian orthoreovirus 1 (Reo1, GenBank: M24734.1, AF378003.1, AF129820.1, AF461682.1, AF490617.1, AF174382.1, M10260.1, L19774.1, M18389.1, X61586.1).

SAMtools v1.9^45^ combined with internal scripts was used to extract essential statistics such as number of mapped reads per virus and coverage. The percentage of identity was derived from the number of variants discovered by BCFtools v1.9^46^.

Results for the different viruses were reported in Excel template files summarizing participant results.

For non-targeted analysis, high-quality reads associated to each sample were processed using a Lab 2 internal data analysis pipeline for adventitious virus detection based on considerations for optimization of high-throughput sequencing bioinformatics pipelines for virus detection^6^.

High-quality reads were aligned using BWA v0.7.17^44^ (options mem -M -a) to a sequence database containing both the *Cricetulus griseus* reference genome (Chinese hamster, CHO, GenBank: GCF_003668045.3) considered as host in this analysis, and the reference virus database, RVDB v23.0^20^, to screen for potential virus reads.

During counter-screening^6^, reads aligned to RVDB were extracted and then aligned to a subset of GenBank v248 sequences using BWA v0.7.17 to assess their origin. The subset of GenBank included only divisions containing mostly reference sequences from bacteria, environment, invertebrates, mammals, phages, plants, primates, rodents, viruses, and vertebrates. Sequences in other GenBank divisions are mostly redundant with selected divisions and are not considered, e.g., gene fragments, artificial sequences, or genome fragments.

All BWA hits having the best equal score were submitted to the Lab 2 taxonomic assignment algorithm that implements a standard Expectation Maximization (EM) algorithm using a mixture model to estimate the proportion of reads generated by each candidate genome or sequence. This approach is similar to TAMER^47^ and to the EM algorithm used in KMCP^48^.

The result of the counter-screening is a taxonomical assignment of the reads consisting mainly in a table with the GenBank accessions of sequences considered as present in the sample tested, their description, their taxonomy, the number of reads mapped to those accessions and the length coverage. Reads assigned to each accession sequence were extracted and realigned only to the assigned accession sequence. Depth of coverage graphs presenting the number of reads covering each nucleotide of reference sequences are then automatically computed for each accession from alignment files.

Virus species were considered as having a high likelihood of presence in the samples if ≥10 reads were observed for a species and ≥300 nucleotides were covered on the species accession sequence capturing the highest number of reads.

Results for viruses that were neither bacteriophages nor virophages were reported in Excel template files collecting participant results.

Additionally, in a follow-up bioinformatics analysis, presence of species was rejected if they met one of the following criteria: stacked reads, only matches less than 33% of original read length.

Lab 3 prepared aliquots of 3 mL for each sample, which were centrifuged at low speed and the resulting supernatant was extracted using QIAamp ccfDNA/RNA Kit (Catalog no. 55184, Qiagen). Total RNA concentration was measured by Qubit 3.0 fluorometer (Thermal Fisher, USA) using Qubit BR assay kit (Catalog no. Q10210, ThermoFisher Scientific). Of note, samples were processed without performing a dedicated preliminary matrix qualification and workflow adaptation, as foreseen by internal workflow procedures. Instead, an internal standard (not matrix-specific) protocol was employed.

Reverse transcription and second-strand synthesis were carried out using an internal workflow based on TruSeq RNA EPH Reagent Tube (Ref. RS-200-1001, Illumina) and TruSeq RNA Library Preparation Kit v2 (Catalog no. RS-122-2001, Illumina). Double-strand cDNA samples were quantified by Qubit 3.0 fluorometer using Qubit dsDNA BR Assay Kit (Catalog no. Q32850, ThermoFisher Scientific). These steps were carried out using a Microlab Star robotic workstation (Hamilton).

Library preparation was performed using Illumina DNA Prep (Cat # 20018705, Illumina −500 ng ds cDNA each sample) and adding indexes (IDT for Illumina DNA/RNA UD Indexes Set A, Catalog no. 20027213, Illumina) according to the manufacturer’s instructions. This step was carried out using a Microlab Star robotic workstation (Hamilton).

Sequencing of fourteen samples was conducted in two runs of Illumina NovaSeq 6000 (seven samples for each run) using the NovaSeq 6000 S2 Reagent Kit v1.5 (300 cycles)  (Cat # 20028314, Illumina). Library sequencing was performed using the following parameters: 2 × 151 cycles paired-end sequencing (according to Illumina’s instructions), pooled library at 1 nM each and PhiX Control v3 Library (1% v/v of pool library spiked) − (Catalog no. FC-110-3001, Illumina).

Demultiplexing of sequencing was performed using the Illumina tool *bcl2fastq* (v. 2.19.1.403)^49^. Read quality was verified with *clumpify* (bbmap v38.96)^39,50^. Read trimming was conducted with *fastp* (v0.23.2)^51^, removing Illumina adapters and index sequences and by removing low-quality bases. Resulting reads were retained only if longer than 50 bp in targeted analysis and if longer than 75 bp in non-targeted analysis and used for subsequent analyses.

For targeted analysis, quality reads were processed using and internal data analysis pipeline for targeted adventitious virus detection. Quality reads were aligned using *bwa mem* (v0.7.17-r1188)^44^ using the option -M against: Human orthopneumovirus (RSV, GenBank: JF920069.1) and Mammalian orthoreovirus 1 (Reo1, GenBank: M24734.1, AF378003.1, AF129820.1, AF461682.1, AF490617.1, AF174382.1, M10260.1, L19774.1, M18389.1, X61586.1) with two different alignment processes.

Coverage metrics on the reference viral sequences were extracted using custom scripts and a set of public tools, including: seqkit (v2.2.0)^52^, bedtools (genomeCoverageBed) (v2.30.0)^53^, infoseq (emboss) (v6.6.0.0)^54^. Results for the different viruses were summarized in Excel files.

For non-targeted analysis, quality reads were processed using and internal data analysis pipeline for non-targeted adventitious virus detection.

During non-targeted analysis, a step of host subtraction was performed in order to remove reads associated with the CHO background. Quality reads were aligned with *Bowtie2* (v2.4.5)^55^ against the reference genomic sequence of *Cricetulus griseus* (Chinese hamster, CHO, GenBank: GCF_003668045.3). Reads mapping against the Chinese hamster genome were removed from the dataset using Samtools (v1.7) and the cigar option -f 13. Sequencing reads were converted back to paired FASTQ file format using bedtools (bamtofastq) (v2.30.0)^53^.

The resulting reads were aligned with *bwa mem* (v0.7.17-r1188)^44^ using the option -M against C-RVDB v23.0. Mapping reads were filtered using Samtools (v1.7) with the cigar option (-F 260).

Coverage metrics on the reference viral sequences were extracted using custom scripts and a set of public tools, including: seqkit (v2.2.0)^52^, bedtools (genomeCoverageBed) (v2.30.0)^53^, infoseq (emboss) (v6.6.0.0)^54^.

Viral sequences were filtered based on signal intensity, minimum length and coverage distribution using pre-defined cutoffs on the number of viral reads, as well as horizontal coverage. In addition, sequences surpassing the defined criteria were further refined using a combination of automatic and manual filters to exclude non-viral/irrelevant hits based on sequence names and taxa. Finally, in a follow-up bioinformatics assessment the detected viral sequences were further evaluated to exclude nonviral hits and reassess the horizontal coverage obtained on partial sequences against the expected viral genome size. Results for the different viruses are summarized in Supplementary Table S1.

### ddPCR assays

ddPCR assays were performed by Lab 1. The extracted nucleic acid samples were the same as for HTS (described above). Three independent assays were done with triplicate samples each and wells with droplet events over 10,000 were analyzed. Samples with ≥10 positive droplets in all triplicates were determined to be positive.

For quantification of RSV GC/mL and determination of the limit of detection (LOD) of the virus spiked in the CHO cell matrix, RT-ddPCRs were performed using the one-step RT-ddPCR advanced kit for probes (Cat # 1864022, BIO-RAD). The RT-ddPCR reaction consisted of: 4X supermix (5.5 µL), 20 U/µL reverse transcriptase (2.2 µL), 300 mM DTT (1.1 µL), 10 µM oligonucleotides of forward and reverse primers (1.98 µL each), 10 µM 5’-FAM/BHQ1-3’ labeled hydrolysis probe (0.55 µL), extracted nucleic acids (2 µL) and nuclease-free water (6.69 µL). The 22 µL reaction was loaded into a 96 -well plate (Cat # 12001925, BIO-RAD) and heat-sealed a foil lid (Cat # 1814040, BIO-RAD) by PX1 PCR plate sealer (BIO-RAD). The reaction mixture was applied to the droplet generator with oil (Cat # 1864110, BIO-RAD) to form droplets of oil-in-water mixture. The plate of mixture was sealed and cycled using a C-1000 touch thermal cycler (BIO-RAD) under the following conditions: 1 h at 50 °C, 10 min at 95 °C, 40 cycles of 30 s at 95°C and 1 min at 57 °C with a ramping rate of 2 °C/s. In the end, 10 min at 98 °C and kept at 4 °C. Finally, the 96-well PCR plate was read on a QX-200 droplet reader (BIO-RAD) using ddPCR droplet reader oil (Cat # 1863004, BIO-RAD) and the number of PCR-positive and -negative droplets for FAM fluorophore was counted.

For Reo1, an equal volume of DMSO (Cat # D2650, Sigma-Aldrich) was added to the sample followed by 95 °C for 3 min and kept on ice for denaturation of dsRNA prior to doing the ddPCR assay. All the other steps were the same as RSV. For the pre-study, primers and probe used for the ddPCR for Reo1 were located in the L2 gene.

For quantification of CHO cell DNA, ddPCR assay for *HMBS* (*Cricetulus griseus* hydroxymethylbilance synthase) gene was developed. Prior to the ddPCR assay, host genome DNA in the test sample, was digested with BsrGI-HF (Cat # R3575L, New England Biolabs Inc.) for 1 h at 37 °C and stored on ice until using it. Each digestion reaction consisted of 100 U of BsrGI-HF, 5 µL of 10X rCutSmart buffer, 5 µL of DNA and 35 µL of nuclease-free H_2_O. The ddPCR supermix for probes (No dUTP) (#1863024, BIO-RAD) contained 2X ddPCR supermix for probes (No dUTP) (11 µL), 10 µM oligonucleotides of forward and reverse primers (1.98 µL each), 10 µM 5’-FAM/BHQ1-3’ labeled hydrolysis probe (0.55 µL), extracted nucleic acids (2 µL) and nuclease-free water (4.49 µL). The amplification conditions were as follows: 10 minutes at 95 °C, followed by 40 cycles of 30 seconds at 95 °C and 1 minute at 56 °C. A subsequent incubation at 98 °C for 10 min was performed to deactivate enyzme activity, followed by storage at 4 °C. All the other steps were the same as described in above section.

The sequences of forward and reverse primers and the probe are indicated in Table 8. ddPCR primers and probes were determined for specificity by blastn analysis against NCBI nr/nt.

## Supplementary information

Supplementary information

## Data Availability

The HTS raw data are available from NCBI BioProject PRJNA1026487 (publicly available).
