# Supplementary material for: Evaluation of high-throughput sequencing for replacing the conventional adventitious virus detection assays used for biologics
Source: NPJ Vaccines. 2025 Dec 23;11:28. doi: 10.1038/s41541-025-01351-2 (PMC12847995; doi:10.1038/s41541-025-01351-2)
Supplement: Supplementary file 1 — Supplementary information [file 41541_2025_1351_MOESM1_ESM.pdf]

Supplementary Table S1. Targeted HTS

		LAB 1							LAB 2							LAB 3						
	Spiking Sample ID	A	C	D	E	F	G	H	A	C	D	E	F	G	H	A	C	D	E	F	G	H
RSV	Total number of reads	292727264	318752214	320160870	318691460	314226034	304283230	293117894	1481755866	1296215744	1304462084	1133323950	1033364940	1289257458	1340556164	1502769062	1073955866	1222235712	1193294902	1071511376	1277117180	1102448340
	Number of quality reads	291580842	316917602	318472152	317358726	312720906	302713998	292125806	1351950988	1155941103	1175342329	1023525142	868712323	1110227605	1177955490	1447766480	1037603984	1179406072	1154116512	1030704836	1232956478	1062753354
	**RSV number of reads**	2	567	289	38	6	0	2	0	520	56	7	0	0	0	0	61	4	0	0	0	0
	**RSV mapped reads parts per million**	0.00685916	1.78910858	0.90745768	0.11973832	0.01918644	0	0.00684637	0	0.44984991	0.04764569	0.00683911	0	0	0	0	0	0	0	0	0	0
	RSV genome coverage (%)	1	52	70	15	3	0	1	0.00%	70.25%	13.83%	2.15%	0.00%	0.00%	0.00%	0	0.05878929	0.00339154	0			
	Total number of reads mapped (coverage/depth)	0.01	2.36	1.89	0.25	0.04	2.36	0.01	0.000	4.085	0.488	0.047	0.000	0.000	0.000	NA	1.61	1	NA	NA	NA	NA
	**Final conclusion**	Positive	Positive	Positive	Positive	Positive	Negative	Positive	Negative	Positive	Positive	Positive	Negative	Negative	Negative	Negative	Positive	Positive	Negative	Negative	Negative	Negative
CHO -K1	**CHO genome number of reads**	233068584	248789358	240923328	236778778	241765055	242197342	217145342	1263794399	1083601404	1102178971	962057309	806842410	1033714428	1095674486	N/A	N/A	N/A	N/A	N/A	N/A	N/A
Reo1	Total number of reads	314495262	310264860	319511584	311877504	310144252	311708068	314139960	1148091814	993084874	1178039918	1219999814	1185532940	1012981934	1274889396	1222536696	913898910	1137301298	1174199128	1010065262	1244683614	1238211190
	Number of quality reads	312825460	308679862	317901376	310335864	308587364	309846060	312777550	981733389	895524585	989416017	1102217731	1047581343	861313359	1073087197	1169296920	876147796	1091327354	1128465426	970866984	1197077464	1190402274
	**Reo1 number of reads**	0	3,840	56	6	0	0	0	0	15	2	0	0	0	0	0	2	0	0	0	0	0
	**Reo1 mapped reads parts per million**	0	12.4400729	0.17615526	0.01933389	0	0	0	0	0.01674996	0.00202139	0	0	0	0	0	0	0	0	0	0	0
	Reo1 genome coverage %	0	25.12	9	2	0	0	0	0.00%	3.41%	0.20%	0.00%	0.00%	0.00%	0.00%	0	0.00228272	0	0	0	0	0
	Total number of reads mapped (coverage/depth)	0	12.12	0.22	0.03	0	12.12	0	0.000	0.083	0.004	0.000	0.000	0.000	0.000	0	0	0	0	0	0	0
	**Final conclusion**	Negative	Positive	Positive	Positive	Negative	Negative	Negative	Negative	Positive	Positive	Negative	Negative	Negative	Negative	Negative	Positive	Negative	Negative	Negative	Negative	Negative
CHO -K1	**CHO genome number of reads**	245672015	231186225	236230969	227385860	229809343	227834142	235356219	923884260	810137626	925285143	1025170428	1013641741	813894884	1004495537	N/A	N/A	N/A	N/A	N/A	N/A	N/A

Supplementary Table S1. Non targeted HTS

		LAB 1								LAB 2								LAB 3						
	Spiking Sample ID	A	C	D	E	F	G	H		A	C	D	E	F	G	H		A	C	D	E	F	G	H
RSV	Total number of reads	292727264	318752214	320160870	318691460	314226034	304283230	293117894		1481755866	1296215744	1304462084	1133323950	1033364940	1289257458	1340556164		1502769062	1073955866	1222235712	1193294902	1071511376	1277117180	1102448340
	Number of quality reads	130671618	146931574	149794560	137362218	146337694	146697092	140239958		1351950988	1155941103	1175342329	1023525142	868712323	1110227605	1177955490		1420646938	1021901974	1159163740	1136190430	1007263696	1209873828	1038552230
	**RSV number of reads**	2	1369	76	12	4	0	0		1	498	55	6	0	1	0		0	44	4	0	0	0	0
	**RSV mapped reads parts per million**	0.015305542	9.317262197	0.507361549	0.087360267	0.027334037	0	0		0.000739672	0.430817797	0.046794877	0.005862093	0	0.000900716	0		0	0.043056967	0.003450764	0	0	0	0
	RSV genome coverage (%)	0.906523	47.3158	7.20412	2.27047	1.31813	0	0		0.20%	69.53%	13.36%	1.48%	0.00%	0.20%	0.00%		0	28.24201813	3.967941138	0	0	0	0
	Total number of reads mapped (coverage/depth)	0.0111673	1.02244	0.0878744	0.0227047	0.0131813	0	0		N/A	N/A	N/A	N/A	N/A	N/A	N/A		N/A	N/A	N/A	N/A	N/A	N/A	N/A
	**Final conclusion**	Negative	Positive	Positive	Positive	Positive	Negative	Negative		Negative	Positive	Positive	Negative	Negative	Negative	Negative		Negative	Positive	Negative	Negative	Negative	Negative	Negative
CHO-K1	**CHO genome number of reads**	N/A	N/A	N/A	N/A	N/A	N/A	N/A		1258954986	1043325779	1063100821	981547461	729868254	981429004	1059438483		1359045330	981298124	1111677364	1088604662	953317180	1150674890	983392558
Reo1	Total number of reads	314495262	310264860	319511584	311877504	310144252	311708068	314139960		1148091814	993084874	1178039918	1219999814	1185532940	1012981934	1274889396		1222536696	913898910	1137301298	1174199128	1010065262	1244683614	1238211190
	Number of quality reads	147269080	136310068	143618858	137292442	144810910	140884224	144491094		981733389	895524585	989416017	1102217731	1047581343	861313359	1073087197		1138629130	855201710	1065419840	1101195414	946258392	1167990184	1165245708
	**Reo1 number of reads**	0	305	23	2	0	0	0		0	19	0	0	0	0	0		0	2	0	0	0	0	0
	**Reo1 mapped reads parts per million**	0	2.237545652	0.1601461	0.014567444	0	0	0		0	0.021216615	0	0	0	0	0		0	0.00233863	0	0	0	0	0
	Reo1 genome coverage %	N/A	N/A	N/A	N/A	N/A	N/A	N/A		0.00%	4.41%	0.00%	0.00%	0.00%	0.00%	0.00%		0	0.999957629	0	0	0	0	0
	Total number of reads mapped (coverage/depth)	N/A	N/A	N/A	N/A	N/A	N/A	N/A		N/A	N/A	N/A	N/A	N/A	N/A	N/A		N/A	N/A	N/A	N/A	N/A	N/A	N/A
	**Final conclusion**	Negative	Positive	Positive	Positive	Negative	Negative	Negative		Negative	Positive	Negative	Negative	Negative	Negative	Negative		Negative	Negative	Negative	Negative	Negative	Negative	Negative
CHO-K1	**CHO genome number of reads**	N/A	N/A	N/A	N/A	N/A	N/A	N/A		851024531	802479740	820242054	1011477923	955457206	738925898	887473669		1078215494	810584006	1008181438	1040016062	895285928	1110280860	1101374986

Supplementary Table S2. In vitro assays

		Lab 2														
		MRC-5					Vero					CHO				
			Guinea Pig					Guinea Pig					Guinea Pig			
			2-8°C		20-25°C			2-8°C		20-25°C			2-8°C		20-25°C	
Spiked virus	IDs	CPE	HAD	HA	HAD	HA	CPE	HAD	HA	HAD	HA	CPE	HAD	HA	HAD	HA
Reo1	A	Negative	Negative	Negative	Negative	Negative	Negative	Negative	Negative	Negative	Negative	Negative	Negative	Negative	Negative	Negative
	B	N/A	N/A	N/A	N/A	N/A	N/A	N/A	N/A	N/A	N/A	N/A	N/A	N/A	N/A	N/A
	C	6/6; D7	N/A	N/A	N/A	N/A	6/6; D7	N/A	N/A	N/A	N/A	6/6; D14	N/A	N/A	N/A	N/A
	D	6/6; D7	N/A	N/A	N/A	N/A	6/6; D7	N/A	N/A	N/A	N/A	6/6; D14	N/A	N/A	N/A	N/A
	E	6/6; D7	N/A	N/A	N/A	N/A	6/6; D7	N/A	N/A	N/A	N/A	6/6; D14	N/A	N/A	N/A	N/A
	F	6/6; D14	N/A	N/A	N/A	N/A	4/6; D7	N/A	N/A	N/A	N/A	6/6; D14	N/A	N/A	N/A	N/A
	G	5/6; D14	N/A	N/A	N/A	N/A	3/6; D14	N/A	N/A	N/A	N/A	6/6; D14	N/A	N/A	N/A	N/A
	H	Negative	Negative	Negative	Negative	Negative	1/6; D14	Negative	Negative	Negative	Negative	6/6; D14	N/A	N/A	N/A	N/A
	NEG ctrl	Negative	Negative	Negative	Negative	Negative	Negative	Negative	Negative	Negative	Negative	Negative	Negative	Negative	Negative	Negative
	POS ctrl	N/A	N/A	N/A	N/A	N/A	N/A	N/A	N/A	N/A	N/A	N/A	N/A	N/A	N/A	N/A
	POS ctrl	N/A	N/A	N/A	N/A	N/A	N/A	N/A	N/A	N/A	N/A	N/A	N/A	N/A	N/A	N/A
	POS ctrl	N/A	N/A	N/A	N/A	N/A	N/A	N/A	N/A	N/A	N/A	N/A	N/A	N/A	N/A	N/A
RSV	A	Negative	Negative	Negative	Negative	Negative	Negative	Negative	Negative	Negative	Negative	Negative	Negative	Negative	Negative	Negative
	B	N/A	N/A	N/A	N/A	N/A	N/A	N/A	N/A	N/A	N/A	N/A	N/A	N/A	N/A	N/A
	C	6/6; D7	N/A	N/A	N/A	N/A	6/6; D7	N/A	N/A	N/A	N/A	6/6; D14	N/A	N/A	N/A	N/A
	D	4/6; D14	N/A	N/A	N/A	N/A	3/6; D7	N/A	N/A	N/A	N/A	6/6; D14	N/A	N/A	N/A	N/A
	E	1/6; D7	N/A	N/A	N/A	N/A	1/6; D7	N/A	N/A	N/A	N/A	6/6; D14	N/A	N/A	N/A	N/A
	F	6/6; D21	N/A	N/A	N/A	N/A	Negative	Negative	Negative	Negative	Negative	Negative	Negative	Negative	Negative	Negative
	G	6/6; D21	N/A	N/A	N/A	N/A	Negative	Negative	Negative	Negative	Negative	6/6; D14	N/A	N/A	N/A	N/A
	H	Negative	Negative	Negative	Negative	Negative	Negative	Negative	Negative	Negative	Negative	Negative	Negative	Negative	Negative	Negative
	NEG ctrl	Negative	Negative	Negative	Negative	Negative	Negative	Negative	Negative	Negative	Negative	Negative	Negative	Negative	Negative	Negative
	POS ctrl	N/A	N/A	N/A	N/A	N/A	N/A	N/A	N/A	N/A	N/A	N/A	N/A	N/A	N/A	N/A
	POS ctrl	N/A	N/A	N/A	N/A	N/A	N/A	N/A	N/A	N/A	N/A	N/A	N/A	N/A	N/A	N/A
	POS ctrl	N/A	N/A	N/A	N/A	N/A	N/A	N/A	N/A	N/A	N/A	N/A	N/A	N/A	N/A	N/A

		Lab 3												
		MRC-5												
			Guinea Pig				Chicken				Rhesus Monnkey			
			2-8°C		34-38°C		2-8°C		34-38°C		2-8°C		34-38°C	
Spiked virus	IDs	CPE	HAD	HA	HAD	HA	HAD	HA	HAD	HA	HAD	HA	HAD	HA
Reo1	A	Negative	Negative	Negative	Negative	Negative	Negative	Negative	Negative	Negative	Negative	Negative	Negative	Negative
	B	N/A	N/A	N/A	N/A	N/A	N/A	N/A	N/A	N/A	N/A	N/A	N/A	N/A
	C	6/6 D14	Negative	Negative	Negative	Negative	Negative	Negative	Negative	Negative	Negative	2/2	Negative	2/2
	D	6/6 D14	Negative	Negative	Negative	Negative	Negative	Negative	Negative	Negative	Negative	2/2	Negative	2/2
	E	6/6 D28	Negative	Negative	Negative	Negative	Negative	Negative	Negative	Negative	Negative	Negative	Negative	Negative
	F	6/6 D28	Negative	Negative	Negative	Negative	Negative	Negative	Negative	Negative	Negative	Negative	Negative	Negative
	G	Negative	Negative	Negative	Negative	Negative	Negative	Negative	Negative	Negative	Negative	Negative	Negative	Negative
	H	Negative	Negative	Negative	Negative	Negative	Negative	Negative	Negative	Negative	Negative	Negative	Negative	Negative
	NEG ctrl	Negative	Negative	Negative	Negative	Negative	Negative	Negative	Negative	Negative	Negative	Negative	Negative	Negative
	POS ctrl	6/6 (D7, D21)	1/2 (D14)	Negative (D14); 2/2 (D28)	Negative	2/2 (D28)	Negative	Negative	Negative	Negative	2/2 (D14, D28)	2/2 (D14, D28)	Negative (D14); 2/2 (D28)	Negative (D14); 2/2 (D28)
	POS ctrl	N/A	N/A	N/A	N/A	N/A	N/A	N/A	N/A	N/A	N/A	N/A	N/A	N/A
	POS ctrl	N/A	N/A	N/A	N/A	N/A	N/A	N/A	N/A	N/A	N/A	N/A	N/A	N/A
RSV	A	Negative	Negative	Negative	Negative	Negative	Negative	Negative	Negative	Negative	Negative	Negative	Negative	Negative
	B	N/A	N/A	N/A	N/A	N/A	N/A	N/A	N/A	N/A	N/A	N/A	N/A	N/A
	C	3/6 D5; 4/6 D7, 5/6 D10-14	Negative	Negative	Negative	Negative	Negative	Negative	Negative	Negative	Negative	Negative	Negative	Negative
	D	1/6 D10-14; 6/6 D18	Negative	Negative	Negative	Negative	Negative	Negative	Negative	Negative	Negative	Negative	Negative	Negative
	E	Negative	Negative	Negative	Negative	Negative	Negative	Negative	Negative	Negative	Negative	Negative	Negative	Negative
	F	Negative	Negative	Negative	Negative	Negative	Negative	Negative	Negative	Negative	Negative	Negative	Negative	Negative
	G	Negative	Negative	Negative	Negative	Negative	Negative	Negative	Negative	Negative	Negative	Negative	Negative	Negative
	H	Negative	Negative	Negative	Negative	Negative	Negative	Negative	Negative	Negative	Negative	Negative	Negative	Negative
	NEG ctrl	Negative	Negative	Negative	Negative	Negative	Negative	Negative	Negative	Negative	Negative	Negative	Negative	Negative
	POS ctrl	6/6 (D7, D18)	Negative	Negative	Negative	Negative	Negative	Negative	Negative	Negative	2/2	2/2	2/2	2/2
	POS ctrl	N/A	N/A	N/A	N/A	N/A	N/A	N/A	N/A	N/A	N/A	N/A	N/A	N/A
	POS ctrl	N/A	N/A	N/A	N/A	N/A	N/A	N/A	N/A	N/A	N/A	N/A	N/A	N/A

		Lab 3												
		Vero												
			Guinea P{ig				Chicken				Rhesus			
			2-8°C		34-38°C		2-8°C		34-38°C		2-8°C		34-38°C	
Spiked virus	IDs	CPE	HAD	HA	HAD	HA	HAD	HA	HAD	HA	HAD	HA	HAD	HA
Reo1	A	Negative	Negative	Negative	Negative	Negative	Negative	Negative	Negative	Negative	Negative	Negative	Negative	Negative
	B	N/A	N/A	N/A	N/A	N/A	N/A	N/A	N/A	N/A	N/A	N/A	N/A	N/A
	C	6/6 D9	Negative	Negative	Negative	Negative	Negative	Negative	Negative	Negative	Negative	Negative	Negative	Negative
	D	6/6 D9	Negative	Negative	Negative	Negative	Negative	Negative	Negative	Negative	Negative	Negative	Negative	Negative
	E	6/6 D14	Negative	Negative	Negative	Negative	Negative	Negative	Negative	Negative	Negative	Negative	Negative	Negative
	F	6/6 D14	Negative	Negative	Negative	Negative	Negative	Negative	Negative	Negative	Negative	Negative	Negative	Negative
	G	6/6 D23	Negative	Negative	Negative	Negative	Negative	Negative	Negative	Negative	Negative	Negative	Negative	Negative
	H	6/6 D23	Negative	Negative	Negative	Negative	Negative	Negative	Negative	Negative	Negative	Negative	Negative	Negative
	NEG ctrl	Negative	Negative	Negative	Negative	Negative	Negative	Negative	Negative	Negative	Negative	Negative	Negative	Negative
	POS ctrl	N/A	N/A	N/A	N/A	N/A	N/A	N/A	N/A	N/A	N/A	N/A	N/A	N/A
	POS ctrl	6/6 (D7, D21)	2/2 (D14, D28)	2/2 (D14, D28)	2/2 (D14, D28)	2/2 (D14, D28)	2/2 (D14, D28)	Negative D14; 2/2 D28	2/2 (D14, D28)	Negative D14; 2/2 D28	2/2 (D14, D28)	2/2 (D14, D28)	2/2 (D14, D28)	Negative D14; 2/2 D28
	POS ctrl	N/A	N/A	N/A	N/A	N/A	N/A	N/A	N/A	N/A	N/A	N/A	N/A	N/A
RSV	A	Negative	Negative	Negative	Negative	Negative	Negative	Negative	Negative	Negative	Negative	Negative	Negative	Negative
	B	N/A	N/A	N/A	N/A	N/A	N/A	N/A	N/A	N/A	N/A	N/A	N/A	N/A
	C	6/6 D10	Negative	Negative	Negative	Negative	Negative	Negative	Negative	Negative	Negative	Negative	Negative	Negative
	D	6/6 D20	Negative	Negative	Negative	Negative	Negative	Negative	Negative	Negative	Negative	Negative	Negative	Negative
	E	Negative	Negative	Negative	Negative	Negative	Negative	Negative	Negative	Negative	Negative	Negative	Negative	Negative
	F	Negative	Negative	Negative	Negative	Negative	Negative	Negative	Negative	Negative	Negative	Negative	Negative	Negative
	G	Negative	Negative	Negative	Negative	Negative	Negative	Negative	Negative	Negative	Negative	Negative	Negative	Negative
	H	Negative	Negative	Negative	Negative	Negative	Negative	Negative	Negative	Negative	Negative	Negative	Negative	Negative
	NEG ctrl	Negative	Negative	Negative	Negative	Negative	Negative	Negative	Negative	Negative	Negative	Negative	Negative	Negative
	POS ctrl	N/A	N/A	N/A	N/A	N/A	N/A	N/A	N/A	N/A	N/A	N/A	N/A	N/A
	POS ctrl	6/6 (D7, D21)	2/2	2/2	2/2	2/2	Negative	Negative	Negative	Negative	2/2	2/2	2/2	2/2
	POS ctrl	N/A	N/A	N/A	N/A	N/A	N/A	N/A	N/A	N/A	N/A	N/A	N/A	N/A

		Lab 3												
		CHO												
			Guinea Pig				Chicken				Rhesus Monkey			
			2-8°C		34-38°C		2-8°C		34-38°C		2-8°C		34-38°C	
Spiked virus	IDs	CPE	HAD	HA	HAD	HA	HAD	HA	HAD	HA	HAD	HA	HAD	HA
Reo1	A	Negative	Negative	Negative	Negative	Negative	Negative	Negative	Negative	Negative	Negative	Negative	Negative	Negative
	B	N/A	N/A	N/A	N/A	N/A	N/A	N/A	N/A	N/A	N/A	N/A	N/A	N/A
	C	6/6 D9	Negative	Negative	Negative	Negative	Negative	Negative	Negative	Negative	Negative	Negative	Negative	Negative
	D	6/6 D9	Negative	Negative	Negative	Negative	Negative	Negative	Negative	Negative	Negative	Negative	Negative	Negative
	E	6/6 D14	Negative	Negative	Negative	Negative	Negative	Negative	Negative	Negative	Negative	Negative	Negative	Negative
	F	6/6 D14	Negative	Negative	Negative	Negative	Negative	Negative	Negative	Negative	Negative	Negative	Negative	Negative
	G	6/6 D28	Negative	Negative	Negative	Negative	Negative	Negative	Negative	Negative	Negative	Negative	Negative	Negative
	H	Negative	Negative	Negative	Negative	Negative	Negative	Negative	Negative	Negative	Negative	Negative	Negative	Negative
	NEG ctrl	Negative	Negative	Negative	Negative	Negative	Negative	Negative	Negative	Negative	Negative	Negative	Negative	Negative
	POS ctrl	N/A	N/A	N/A	N/A	N/A	N/A	N/A	N/A	N/A	N/A	N/A	N/A	N/A
	POS ctrl	N/A	N/A	N/A	N/A	N/A	N/A	N/A	N/A	N/A	N/A	N/A	N/A	N/A
	POS ctrl	6/6 (D7); 3/6 (D23) 6/6 (25	2/2 (D14; D28)	2/2 (D14)	2/2 (D28)	2/2 (D14, D28)	1/2 (D14, D28)	Negative	Negative	Negative	2/2 (D14, D28)	2/2 (D14)	2/2 (D28)	2/2 (D14, D28)
RSV	A	Negative	Negative	Negative	Negative	Negative	Negative	Negative	Negative	Negative	Negative	Negative	Negative	Negative
	B	N/A	N/A	N/A	N/A	N/A	N/A	N/A	N/A	N/A	N/A	N/A	N/A	N/A
	C	Negative	Negative	Negative	Negative	Negative	Negative	Negative	Negative	Negative	Negative	Negative	Negative	Negative
	D	Negative	Negative	Negative	Negative	Negative	Negative	Negative	Negative	Negative	Negative	Negative	Negative	Negative
	E	Negative	Negative	Negative	Negative	Negative	Negative	Negative	Negative	Negative	Negative	Negative	Negative	Negative
	F	Negative	Negative	Negative	Negative	Negative	Negative	Negative	Negative	Negative	Negative	Negative	Negative	Negative
	G	Negative	Negative	Negative	Negative	Negative	Negative	Negative	Negative	Negative	Negative	Negative	Negative	Negative
	H	Negative	Negative	Negative	Negative	Negative	Negative	Negative	Negative	Negative	Negative	Negative	Negative	Negative
	NEG ctrl	Negative	Negative	Negative	Negative	Negative	Negative	Negative	Negative	Negative	Negative	Negative	Negative	Negative
	POS ctrl	N/A	N/A	N/A	N/A	N/A	N/A	N/A	N/A	N/A	N/A	N/A	N/A	N/A
	POS ctrl	N/A	N/A	N/A	N/A	N/A	N/A	N/A	N/A	N/A	N/A	N/A	N/A	N/A
	POS ctrl	6/6 (D6); 6/6 (D19)	2/2	2/2	2/2	2/2	Negative	Negative	Negative	Negative	2/2	2/2	2/2	2/2

Supplementary Table S3. List of CHO rRNAs for removal of host reads by Lab 1

NCBI Accession Number	Description	Size
XM_027418737	PREDICTED: Cricetulus griseus ribosomal RNA processing 15 homolog (Rrp15), mRNA.	1202
XM_027414413	PREDICTED: Cricetulus griseus ribosomal RNA processing 9, U3 small nucleolar RNA binding protein (Rrp9), mRNA.	1570
XM_027404958	PREDICTED: Cricetulus griseus ribosomal RNA processing 8 (Rrp8), transcript variant X4, mRNA.	2301
XR_003483234	PREDICTED: Cricetulus griseus ribosomal RNA processing 8 (Rrp8), transcript variant X3, misc_RNA.	1776
XR_004769111	PREDICTED: Cricetulus griseus ribosomal RNA processing 8 (Rrp8), transcript variant X2, misc_RNA.	1918
XM_027404957	PREDICTED: Cricetulus griseus ribosomal RNA processing 8 (Rrp8), transcript variant X1, mRNA.	2302
XM_027407320	PREDICTED: Cricetulus griseus ribosomal RNA processing 12 homolog (Rrp12), transcript variant X2, mRNA.	4348
XM_027396106	PREDICTED: Cricetulus griseus ribosomal RNA processing 7 homolog A (Rrp7a), mRNA.	2129
XM_035451809	PREDICTED: Cricetulus griseus ribosomal RNA processing 1B (Rrp1b), transcript variant X9, mRNA.	4595
XR_004772080	PREDICTED: Cricetulus griseus ribosomal RNA processing 1B (Rrp1b), transcript variant X8, misc_RNA.	2895
XR_003481005	PREDICTED: Cricetulus griseus ribosomal RNA processing 1B (Rrp1b), transcript variant X7, misc_RNA.	4171
XR_003481004	PREDICTED: Cricetulus griseus ribosomal RNA processing 1B (Rrp1b), transcript variant X6, misc_RNA.	3234
XM_027394358	PREDICTED: Cricetulus griseus ribosomal RNA processing 1B (Rrp1b), transcript variant X5, mRNA.	4510
XM_035451692	PREDICTED: Cricetulus griseus ribosomal RNA processing 36 (Rrp36), transcript variant X3, mRNA.	1284
XM_035451691	PREDICTED: Cricetulus griseus ribosomal RNA processing 36 (Rrp36), transcript variant X2, mRNA.	1176
XM_035451690	PREDICTED: Cricetulus griseus ribosomal RNA processing 36 (Rrp36), transcript variant X1, mRNA.	1179
XM_027394355	PREDICTED: Cricetulus griseus ribosomal RNA processing 1 (Rrp1), transcript variant X2, mRNA.	1724
XM_035451127	PREDICTED: Cricetulus griseus ribosomal RNA adenine dimethylase domain containing 1 (Rrnad1), transcript variant X20, mRNA.	1748
XM_035451126	PREDICTED: Cricetulus griseus ribosomal RNA adenine dimethylase domain containing 1 (Rrnad1), transcript variant X19, mRNA.	1514
XM_027393068	PREDICTED: Cricetulus griseus ribosomal RNA adenine dimethylase domain containing 1 (Rrnad1), transcript variant X18, mRNA.	2687
XM_035451125	PREDICTED: Cricetulus griseus ribosomal RNA adenine dimethylase domain containing 1 (Rrnad1), transcript variant X17, mRNA.	2727
XM_035451124	PREDICTED: Cricetulus griseus ribosomal RNA adenine dimethylase domain containing 1 (Rrnad1), transcript variant X16, mRNA.	2648
XM_035451123	PREDICTED: Cricetulus griseus ribosomal RNA adenine dimethylase domain containing 1 (Rrnad1), transcript variant X15, mRNA.	1793
XM_035451122	PREDICTED: Cricetulus griseus ribosomal RNA adenine dimethylase domain containing 1 (Rrnad1), transcript variant X13, mRNA.	2528
XM_027393066	PREDICTED: Cricetulus griseus ribosomal RNA adenine dimethylase domain containing 1 (Rrnad1), transcript variant X12, mRNA.	1934
XM_027393065	PREDICTED: Cricetulus griseus ribosomal RNA adenine dimethylase domain containing 1 (Rrnad1), transcript variant X11, mRNA.	2708
XM_027393064	PREDICTED: Cricetulus griseus ribosomal RNA adenine dimethylase domain containing 1 (Rrnad1), transcript variant X10, mRNA.	1974
XM_035451121	PREDICTED: Cricetulus griseus ribosomal RNA adenine dimethylase domain containing 1 (Rrnad1), transcript variant X9, mRNA.	2748
XM_027393063	PREDICTED: Cricetulus griseus ribosomal RNA adenine dimethylase domain containing 1 (Rrnad1), transcript variant X8, mRNA.	1853
XM_027393061	PREDICTED: Cricetulus griseus ribosomal RNA adenine dimethylase domain containing 1 (Rrnad1), transcript variant X6, mRNA.	1946

XM_035451120	PREDICTED: Cricetulus griseus ribosomal RNA adenine dimethylase domain containing 1 (Rrnad1), transcript variant X5, mRNA.	1979
XM_035451119	PREDICTED: Cricetulus griseus ribosomal RNA adenine dimethylase domain containing 1 (Rrnad1), transcript variant X4, mRNA.	2067
XM_027393057	PREDICTED: Cricetulus griseus ribosomal RNA adenine dimethylase domain containing 1 (Rrnad1), transcript variant X3, mRNA.	1832
XM_035451118	PREDICTED: Cricetulus griseus ribosomal RNA adenine dimethylase domain containing 1 (Rrnad1), transcript variant X2, mRNA.	2865
XM_027385942	PREDICTED: Cricetulus griseus ribosomal RNA processing 1 (Rrp1), transcript variant X2, mRNA.	1724
XM_003515068	PREDICTED: Cricetulus griseus ribosomal RNA processing 1 (Rrp1), transcript variant X1, mRNA.	1783
XM_003512858	PREDICTED: Cricetulus griseus ribosomal RNA processing 8 (Rrp8), transcript variant X4, mRNA.	2299
XR_483896	PREDICTED: Cricetulus griseus ribosomal RNA processing 8 (Rrp8), transcript variant X3, misc_RNA.	1774
XR_004767410	PREDICTED: Cricetulus griseus ribosomal RNA processing 8 (Rrp8), transcript variant X2, misc_RNA.	1916
XM_007653264	PREDICTED: Cricetulus griseus ribosomal RNA processing 8 (Rrp8), transcript variant X1, mRNA.	2300
XR_004775160	PREDICTED: Cricetulus griseus ribosomal RNA processing 1B (Rrp1b), transcript variant X4, misc_RNA.	2882
XR_001728443	PREDICTED: Cricetulus griseus ribosomal RNA processing 1B (Rrp1b), transcript variant X3, misc_RNA.	4158
XR_003478048	PREDICTED: Cricetulus griseus ribosomal RNA processing 1B (Rrp1b), transcript variant X2, misc_RNA.	3221
XM_007652154	PREDICTED: Cricetulus griseus ribosomal RNA processing 1B (Rrp1b), transcript variant X1, mRNA.	4497
XM_003508798	PREDICTED: Cricetulus griseus ribosomal RNA processing 7 homolog A (Rrp7a), mRNA.	2148
XM_035458099	PREDICTED: Cricetulus griseus ribosomal RNA processing 36 (Rrp36), transcript variant X3, mRNA.	1281
XM_035458098	PREDICTED: Cricetulus griseus ribosomal RNA processing 36 (Rrp36), transcript variant X2, mRNA.	1189
XM_035458097	PREDICTED: Cricetulus griseus ribosomal RNA processing 36 (Rrp36), transcript variant X1, mRNA.	1192
XM_003502902	PREDICTED: Cricetulus griseus ribosomal RNA adenine dimethylase domain containing 1 (Rrnad1), transcript variant X1, mRNA.	2516
XM_003500367	PREDICTED: Cricetulus griseus ribosomal RNA processing 9, U3 small nucleolar RNA binding protein (Rrp9), mRNA.	1570
XM_016967513	PREDICTED: Cricetulus griseus ribosomal RNA processing 12 homolog (Rrp12), transcript variant X1, mRNA.	4343
XM_003494975	PREDICTED: Cricetulus griseus ribosomal RNA processing 15 homolog (Rrp15), mRNA.	1202
XR_003488776	PREDICTED: Cricetulus griseus 28S ribosomal RNA (LOC113839001), rRNA.	4639
XR_003488775	PREDICTED: Cricetulus griseus 5.8S ribosomal RNA (LOC113839000), rRNA.	153
XR_003488597	PREDICTED: Cricetulus griseus 28S ribosomal RNA (LOC113838089), rRNA.	4677
XR_003488596	PREDICTED: Cricetulus griseus 5.8S ribosomal RNA (LOC113838088), rRNA.	153
XR_003488590	PREDICTED: Cricetulus griseus 28S ribosomal RNA (LOC113838061), rRNA.	4759
XR_003488589	PREDICTED: Cricetulus griseus 5.8S ribosomal RNA (LOC113838060), rRNA.	153
XR_003487346	PREDICTED: Cricetulus griseus 5S ribosomal RNA (LOC113834995), rRNA.	119
XM_027394354	PREDICTED: Cricetulus griseus ribosomal RNA processing 1 (Rrp1), transcript variant X1, mRNA.	1783
XR_003484453	PREDICTED: Cricetulus griseus 5S ribosomal RNA (LOC113835150), rRNA.	119
XR_003484452	PREDICTED: Cricetulus griseus 5S ribosomal RNA (LOC113835149), rRNA.	119

XR_003484451	PREDICTED: Cricetulus griseus 5S ribosomal RNA (LOC113835148), rRNA.	119
XR_003484446	PREDICTED: Cricetulus griseus 5S ribosomal RNA (LOC113835146), rRNA.	119
XM_027393067	PREDICTED: Cricetulus griseus ribosomal RNA adenine dimethylase domain containing 1 (Rrnad1), transcript variant X14, mRNA.	1559
XM_027393062	PREDICTED: Cricetulus griseus ribosomal RNA adenine dimethylase domain containing 1 (Rrnad1), transcript variant X7, mRNA.	1619
XR_003487068	PREDICTED: Cricetulus griseus 5S ribosomal RNA (LOC113836661), rRNA.	119
XR_003484283	PREDICTED: Cricetulus griseus 5S ribosomal RNA (LOC113830952), rRNA.	119
XR_003488144	PREDICTED: Cricetulus griseus 5S ribosomal RNA (LOC113831200), rRNA.	119
XR_003483221	PREDICTED: Cricetulus griseus 5S ribosomal RNA (LOC113834594), rRNA.	119
XR_003483220	PREDICTED: Cricetulus griseus 5S ribosomal RNA (LOC113834593), rRNA.	119
XR_003483219	PREDICTED: Cricetulus griseus 5S ribosomal RNA (LOC113834592), rRNA.	119
XR_003483217	PREDICTED: Cricetulus griseus 5S ribosomal RNA (LOC113834591), rRNA.	119
XR_003478492	PREDICTED: Cricetulus griseus 5S ribosomal RNA (LOC113831200), rRNA.	119
XR_003478229	PREDICTED: Cricetulus griseus 5S ribosomal RNA (LOC113830952), rRNA.	119
XR_003484095	PREDICTED: Cricetulus griseus 5S ribosomal RNA (LOC113834995), rRNA.	119
NR_046263	Cricetulus griseus 45S ribosomal RNA (Rn45s), ribosomal RNA.	8240
AY012116	Cricetulus griseus 12S ribosomal RNA gene, partial sequence; and tRNA-Val gene, complete sequence; mitochondrial.	1023
AY011148	Cricetulus griseus 16S ribosomal RNA gene, partial sequence; mitochondrial.	808
NR_045212	Cricetulus griseus 28S ribosomal RNA (Rn28s1), ribosomal RNA.	1929
NR_045133	Cricetulus griseus 5.8S ribosomal RNA (Rn5-8s), ribosomal RNA.	157
NR_045132	Cricetulus griseus 18S ribosomal RNA (Rn18s), ribosomal RNA.	1877
DQ334846	Cricetulus griseus cell-line CCL-14 16S ribosomal RNA gene, partial sequence; mitochondrial.	1032
DQ334844	Cricetulus griseus cell-line CRL-10154 16S ribosomal RNA gene, partial sequence; mitochondrial.	1036
AF373329	Cricetulus griseus clone pRB-6U-22 28S ribosomal RNA gene, partial sequence.	156
DQ235090	Cricetulus griseus external transcribed spacer, 18S ribosomal RNA gene, internal transcribed spacer 1, and 5.8S ribosomal RNA gene, complete sequence; and internal transcribed spacer 2, partial	15029
AY390526	Cricetulus griseus external transcribed spacer, 18S ribosomal RNA gene, internal transcribed spacer 1, 5.8S ribosomal RNA gene, and internal transcribed spacer 2, complete sequence; and 28S ribosomal RNA gene, partial sequence.	8240
M14803	Chinese hamster ribosomal RNA transcription initiation site.	770
EU518200	Cricetulus griseus strain inbred SYB1 16S ribosomal RNA gene, partial sequence; mitochondrial.	652
EU518199	Cricetulus griseus strain inbred SYB1 12S ribosomal RNA gene, partial sequence; mitochondrial.	685
AF373330	Cricetulus griseus clone pRB-6U-121 28S ribosomal RNA gene, partial sequence.	266
